# Advancing remote sensing and machine learning‐driven frameworks for groundwater withdrawal estimation in Arizona: Linking land subsidence to groundwater withdrawals

**DOI:** 10.1002/hyp.14757

**Published:** 2022-11-14

**Authors:** Sayantan Majumdar, Ryan Smith, Brian D. Conway, Venkataraman Lakshmi

**Affiliations:** ^1^ Department of Civil and Environmental Engineering Colorado State University Fort Collins Colorado USA; ^2^ Arizona Department of Water Resources Phoenix Arizona USA; ^3^ Department of Engineering Systems and Environment University of Virginia Charlottesville Virginia USA

**Keywords:** estimation and forecasting, geospatial, groundwater hydrology, InSAR, land subsidence, machine learning, remote sensing, time series analysis

## Abstract

Groundwater plays a crucial role in sustaining global food security but is being over‐exploited in many basins of the world. Despite its importance and finite availability, local‐scale monitoring of groundwater withdrawals required for sustainable water management practices is not carried out in most countries, including the United States. In this study, we combine publicly available datasets into a machine learning framework for estimating groundwater withdrawals over the state of Arizona. Here we include evapotranspiration, precipitation, crop coefficients, land use, annual discharge, well density, and watershed stress metrics for our predictions. We employ random forests to predict groundwater withdrawals from 2002 to 2020 at a 2 km spatial resolution using in situ groundwater withdrawal data available for Arizona Active Management Areas (AMA) and Irrigation Non‐Expansion Areas (INA) from 2002 to 2009 for training and 2010–2020 for validating the model respectively. The results show high training ($R^{2}\approx 0.9$) and good testing ($R^{2}\approx 0.7$) scores with normalized mean absolute error (NMAE) ≈ 0.62 and normalized root mean square error (NRMSE) ≈ 2.34 for the AMA/INA region. Using this method, we spatially extrapolate the existing groundwater withdrawal estimates to the entire state and observe the co‐occurrence of both groundwater withdrawals and land subsidence in South‐Central and Southern Arizona. Our model predicts groundwater withdrawals in regions where production wells are present on agricultural lands and subsidence is observed from Interferometric Synthetic Aperture Radar (InSAR), but withdrawals are not monitored. By performing a comparative analysis over these regions using the predicted groundwater withdrawals and InSAR‐based land subsidence estimates, we observe a varying degree of subsidence for similar volumes of withdrawals in different basins. The performance of our model on validation datasets and its favourable comparison with independent water use proxies such as InSAR demonstrate the effectiveness and extensibility of our combined remote sensing and machine learning‐based approach.

## Graphical abstract

Groundwater plays a crucial role in sustaining global food security but is being over‐exploited in many basins of the world and the extent of water use is unknown in most regions. In this work, we successfully demonstrated a method to estimate groundwater use by integrating satellite datasets with machine learning. This work could aid water managers in putting sustainability efforts into practice.

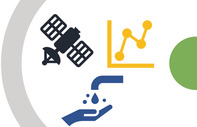

## 1 INTRODUCTION

Constituting ~30% of total global freshwater reserves (Schneider et al., 2011), groundwater is the largest source of Earth's liquid freshwater and is an essential resource responsible for sustaining the water‐food‐energy nexus (Smajgl et al., 2016). With nearly half of the global drinking water being supplied by groundwater and the ever‐increasing demands for agricultural products primarily driven by the rising global population and dietary shifts, groundwater is being rapidly depleted (Margat & van der Gun, 2013). Specifically, since the latter half of the 20th century, demands for non‐renewable groundwater reserves have tripled, providing about 20% of global irrigation water (Wada et al., 2012). Due to these large demands/dependence on groundwater, several key agricultural regions around the world are encountering the negative impacts of groundwater overuse, which include permanent aquifer depletion (Butler et al., 2018; Cao et al., 2013; Faunt et al., 2016; Rodell et al., 2009; Scanlon et al., 2012; Shekhar et al., 2020; Smith et al., 2017; Tiwari et al., 2009), land subsidence (Galloway & Burbey, 2011; Herrera‐García et al., 2021; Smith & Li, 2021; Smith & Majumdar, 2020), and water contamination (Costall et al., 2020; Erban et al., 2013; Goebel et al., 2017; Gottschalk et al., 2020; Levy et al., 2021; Smith et al., 2018). Despite such pressing challenges, groundwater withdrawals (also known as extraction or pumping) are not monitored in most areas at a scale suitable for implementing sustainable groundwater management practices (Foster et al., 2020). As a result, robust and effective methods are needed to reliably quantify groundwater use for aiding efforts to better manage this heavily stressed resource (Majumdar et al., 2020).

Currently, established methods to estimate groundwater withdrawals depend on agricultural water demand, which is usually calculated using evapotranspiration and soil models along with surface water availability. For example, the Central Valley Hydrologic Model (CVHM) developed by Faunt (2009) uses the MODFLOW FMP package (Schmid, 2004) to simulate water demand. This is a sophisticated approach that incorporates land use data and an evapotranspiration model relying on temperature, crop type, precipitation, and root depth. CVHM then apportions the remaining water demand to groundwater withdrawals after accounting for surface water demand using known surface water availability data. The Integrated Water Flow Model (IWFM) (Dogrul, Brush, & Kadir, 2016; Dogrul, Schmid, et al., 2016) is a similar approach and has been used to simulate the hydrologic cycle, including agricultural water demand. Cao et al. (2013) also developed a flow model using MODFLOW to simulate the spatiotemporal variability of groundwater depletion related to groundwater pumping in the North China Plain. The Arizona Department of Water Resources (ADWR) has published several reports on similar groundwater flow models simulating water supply and future water demands for various regions in Arizona (ADWR, 2018b). The Aquaculture and Irrigation Water‐Use Model (Wilson, 2021) developed for the Mississippi Alluvial Plain region combines remotely sensed land use data, in situ pumping data, and look‐up tables to estimate crop‐specific groundwater use at local scales (~1.6 km). Although these models have been successfully applied and have produced added insights into the groundwater flow regime, calibrating and extending them to other regions or large geographical areas can be extremely expensive due to the inherent complexity and number of parameters involved.

The growing availability of remote sensing data sets, gridded hydrometeorological data, and digitized hydrography data has enabled us to monitor large‐scale regions for various hydrologic applications (Frappart & Bourrel, 2018; Lakshmi et al., 2018; Leidner & Buchanan, 2018). Total water storage—GRACE (Gravity Recovery and Climate Experiment) and GRACE‐FO (GRACE‐Follow On) (Nie et al., 2018), terrestrial evapotranspiration—SSEBop (Operational Simplified Surface Energy Balance) (Senay et al., 2013), precipitation—PRISM (Parameter‐elevation Regressions on Elevation Slopes Model) (Daly et al., 2008), land use—USDA‐NASS (United States Department of Agriculture‐National Agricultural Statistics Service) (Boryan et al., 2011), and hydrography—NHD (National Hydrography Dataset) (Simley, 2008) are some of the widely used openly available data sets over the conterminous United States (US).

The individual use of these data sets for estimating groundwater storage fluxes has been reported in several studies. Rodell et al. (2007), Rodell et al. (2009), and Famiglietti et al. (2011) used GRACE‐derived total water storage changes to estimate groundwater fluxes after subtracting the components of snow water, surface water, and soil moisture. Although GRACE and GRACE‐FO data are helpful for basin‐ or continental‐scale studies, their application to local‐scale groundwater storage change estimation is hindered by the coarse resolution (~400 km). In addition, the application of remote sensing derived land use data sets to estimate irrigated area is becoming more common, but historically, these have not been directly related to groundwater withdrawals (Deines et al., 2017, 2020, 2021; Ozdogan & Gutman, 2008). Moreover, land subsidence estimates from spaceborne Interferometric Synthetic Aperture Radar (InSAR) techniques have been used in some studies to estimate groundwater storage changes at high spatial resolutions (~100 m) (Chaussard et al., 2014; J. Chen et al., 2017; Hoffmann et al., 2001; M. M. Miller & Shirzaei, 2015; Reeves et al., 2011; Smith et al., 2017) but these are typically restricted to specific regions having confined or semi‐confined aquifers and highly compressible sediments (Smith & Majumdar, 2020). Despite several well‐established research efforts in these fields, combined use of these data sets to estimate groundwater fluxes is rare.

The varying spatial and temporal resolutions of different remote sensing products pose a challenge for integrating them to estimate water balance components (Tamayo‐Mas et al., 2018). Additionally, to directly estimate groundwater withdrawals using a water balance approach, we require knowledge of several essential variables such as surface water withdrawals, groundwater recharge, and inflow/outflow, which are often complicated and sometimes impossible to obtain. In this context, it has been shown that, in many cases, the accuracy of existing water balance estimates is limited to some extent because of spatial bias (Hashemi et al., 2017). Furthermore, traditional approaches involving physical models tend to become overly complex and computationally expensive, especially when applied to large regions, when various remote sensing products are utilized (Becker et al., 2019; Faunt, 2009; Moeck et al., 2018; Seibert et al., 2019; Tamayo‐Mas et al., 2018).

Our earlier research in Kansas, consisting of semi‐arid to sub‐humid climatic regions (Majumdar et al., 2020), is possibly the earliest work on integrating multitemporal remote sensing products to estimate local‐scale (5 km) groundwater withdrawals. Similar to Majumdar et al. (2020), in this work, we develop an approach to estimate groundwater withdrawals at a higher resolution (2 km) by utilizing a diverse collection of remote sensing and gridded hydrometeorological products that relate to the different water balance components. Here, we test this approach in Arizona, which experiences arid to semi‐arid climates (ADWR, 2020c), and perform sensitivity analysis to different resolutions. We also compare our estimates of groundwater withdrawals with subsidence data from InSAR, marking the first study to compare withdrawals and subsidence over such a large ($\sim 3.0\times 10^{5}$ km^2^) region.

As in Majumdar et al. (2020), we apply random forests (RF) (Belgiu & Drăguţ, 2016; Breiman, 2001), a widely popular machine learning algorithm, to obtain local‐scale estimates of groundwater withdrawals over the state of Arizona for the period 2002–2020. The model is calibrated and validated using in situ groundwater pumping data available from the ADWR data archive (ADWR, 2020a). The remote sensing products incorporated in our study include SSEBop evapotranspiration (Senay et al., 2013) and land use from USDA‐NASS (Boryan et al., 2011). Additionally, we use the PRISM gridded precipitation data (Daly et al., 2008) and crop coefficients (CC) (Allen et al., 1998) along with well density (ADWR, 2020a) and watershed water stress indices developed by Smith and Majumdar (2020) as predictors for our model. We further use the NHD (Simley, 2008) to derive rasterized canal data which is ingested as a model predictor and accounts for imported surface water from the Colorado River (ADWR, 1983; CAP, 2022). We also cross‐compare the estimated groundwater withdrawals and sediment thickness data obtained from the US Geological Survey (USGS) (Shah & Boyd, 2018) with InSAR‐derived land subsidence available from ADWR (2019) to provide insights into the causes of significant land subsidence in Southern and South‐Central parts of Arizona (ADWR, 2019; Conway, 2016).

The remainder of this paper is divided into four sections. We first discuss the characteristics of the study area, data sets used, and the details of our workflow (Section 2), followed by an analysis of the results (Section 3). Finally, we conclude with a discussion of our findings, their implications, and their applicability to other areas (Sections 4 and 5).

## 2 STUDY AREA, DATA, AND METHODS

### 2.1 Study area

There are two regionally extensive and predominantly used aquifer systems in Arizona, the Basin and Range Aquifer, covering the southern and western parts, and the Colorado Plateau aquifers, which cover the northern and eastern parts of the state (Anderson et al., 2006). Groundwater is extensively used in the agriculture sector, which grows year‐round crops such as cotton, alfalfa, wheat, and several other specialty crops (AZDA, 2019). Arizona experiences arid and semi‐arid climates with extreme precipitation variability and is currently in its 26th year of a long‐term drought (ADWR, 2020c). With increasing demands for freshwater resources and ongoing drought conditions, groundwater resources are being heavily stressed, primarily in Southern and South‐Central Arizona, where there is limited surface water availability, and pumping is generally from thick, unconsolidated aquifers, thereby leading to significant land subsidence (ADWR, 2019, 2020b; Anderson et al., 2006; Conway, 2016).

**FIGURE 1:**
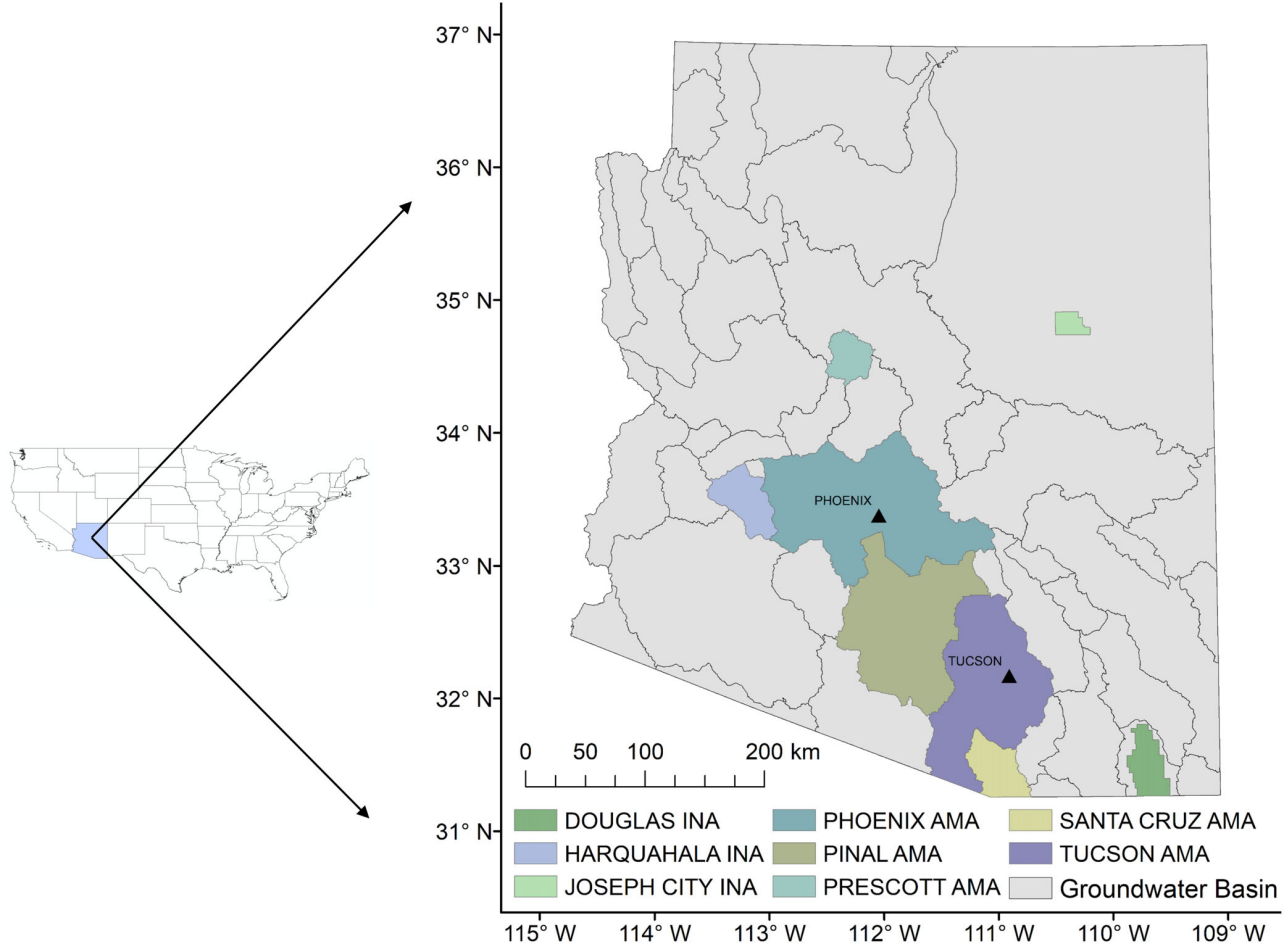
Groundwater basin map of Arizona highlighting the AMA/INA region and the cities of Phoenix and Tucson

Following the 1980 Arizona Groundwater Management Act (GMA) (ADWR, 2020b), areas exhibiting substantial groundwater reliance were identified and designated as Active Management Areas (AMAs). These include Prescott, Phoenix, Pinal, Tucson, and Santa Cruz. Additionally, rural farming areas (Joseph City, Douglas, and Harquahala) experiencing groundwater overdraft issues of lesser severity than AMAs were designated as Irrigation Non‐Expansion Areas (INAs). These AMAs and INAs are shown in Figure 1. AMAs have the highest level of regulation and require that no new irrigation land will be developed, that all major use wells be metered and reported, and that these reports may be audited. AMAs have the additional stipulation of requiring a groundwater management plan, which in most cases requires that water coming into the aquifer is at least as much as water being withdrawn (termed safe yield) by 2025 (ADWR, 2020b). While INAs are not as heavily regulated, they also require that no new irrigation land be developed, and that water use from all major use wells is metered and reported annually (ADWR, 2020b).

Compared to Kansas, where the climate varies from semi‐arid in the west to sub‐humid in the east (Lin et al., 2017), Arizona experiences an arid to semi‐arid climate (ADWR, 2020c). Kansas primarily draws its groundwater from the High Plains Aquifer, which consists of unconsolidated blanket‐like sand and gravel (alluvial deposits) (A. J. Miller & Appel, 1997). However, in Arizona, groundwater pumpage is predominantly from the unconsolidated basin‐fill or valley‐fill aquifer (Basin and Range Aquifer) and from the poorly‐to‐well consolidated sedimentary aquifers (Colorado Plateau aquifers) (Robson & Banta, 1995). Moreover, Arizona produces a much wider variety of crops, for example, citrus fruits, leafy vegetables, nuts, and other specialty crops, in addition to wheat, corn, and cotton (which are also grown in Kansas) (AZDA, 2019; Kansas Department of Agriculture, 2020).

Owing to these considerable differences in aquifer properties, climatic characteristics, and crop production compared to our earlier work in Kansas (Majumdar et al., 2020) and the availability of sizeable in situ groundwater withdrawal data from ADWR, we chose Arizona as the test site for extending our earlier approach. Moreover, understanding the links between groundwater pumping and land subsidence was another motivation behind selecting this region.

### 2.2 Data

In this study, we use a collection of spatio‐temporal variables and temporally static data sets. The spatio‐temporal data sets (2002–2020) include SSEBop evapotranspiration (ET), PRISM‐derived precipitation (P), watershed stress calculated using average precipitation (WS_PA_), and watershed stress calculated using average precipitated adjusted with average evapotranspiration (WS_PA/EA_). Regarding the temporally static data sets, we include the mean USDA‐NASS Cropland Data Layer (CDL) land use densities (averaged from 2008 to 2020), CC, canal data (canals connected to the Colorado River), and well density map (WD). The in situ groundwater withdrawal data were obtained from ADWR (2020a). A summary of these data sets is provided in the following paragraphs.

In our research, we used the cumulative monthly SSEBop ET and PRISM precipitation data for the year‐round growing season (January–December) from 2002 to 2020. The SSEBop ET data (Senay et al., 2013) are computed using the Moderate Resolution Imaging Spectroradiometer (MODIS) land surface temperature product (Wan, 2013) and model‐assimilated weather fields. The data sets are available at 1 km spatial resolution over the conterminous US (CONUS) from daily to seasonal time scales. We incorporated the gridded PRISM precipitation product available from the PRISM group (Daly et al., 2008). The daily or monthly precipitation estimates, which are available CONUS‐wide at a 4‐km spatial resolution, are computed using weighted spatial regression methods wherein the weights are derived from various physiographic entities, including topography and location (Daly et al., 2008).

In addition, we use two watershed water stress indices (Smith & Majumdar, 2020) using surface watershed shapefiles obtained from ADWR (2021a) and USDA‐NASS CDL land use. WS_PA_ (watershed stress index using average precipitation) given by Equation (1) is calculated as in Smith and Majumdar (2020). WS_PA/EA_ (WS_PA_ adjusted for ET), provided by Equation (2), is a slight variant of WS_PA_ and accounts for the average ET within a particular watershed. These indices indicate surface water availability per agricultural or urban land area, wherein the underlying assumption is that there are increased water demands in these developed land areas (Smith & Majumdar, 2020). Accordingly, the positive and negative values of these metrics indicate lesser and more water stress, respectively.

(1) WSPA=Pavg1000−LUPavg1000+LU

(2) $${\mathrm{WS}}_{\mathrm{PA}/\mathrm{EA}}=\frac{\frac{P_{\mathrm{avg}}-{\mathrm{ET}}_{\mathrm{avg}}}{1000}-\mathrm{LU}}{\frac{P_{\mathrm{avg}}-{\mathrm{ET}}_{\mathrm{avg}}}{1000}+\mathrm{LU}}$$

where, LU=C+U2; *C* = number of cropland pixels within a watershed; *U* = number of urban pixels within a watershed; *P*_avg_ = average precipitation (in mm) in a watershed; ET_avg_ = average evapotranspiration (in mm) in a watershed.

In this work, we use the USDA‐NASS CDL (Boryan et al., 2011) data sets from 2008 to 2020, which are annual crop‐specific land cover data layers developed using Landsat and ground data, available at 30 m spatial resolution over the CONUS. Moreover, we incorporate the mid‐seasonal stage CC (Allen et al., 1998) as a proxy for irrigated agriculture (e.g., corn has a higher likelihood of being irrigated than sorghum).

The ADWR Groundwater Site Inventory (GWSI) database (ADWR, 2021a) contains well information (location, well depth, etc.) for the state. We use it to obtain a well density map that provides the number of wells in each pixel. In addition, we incorporate the ADWR groundwater basin shapefile (ADWR, 2021a), USGS sediment thickness data (Shah & Boyd, 2018), and the InSAR‐derived land subsidence estimates provided by ADWR (2021a) for determining the relationship between groundwater withdrawals and land subsidence. The sediment thickness data set, which represents the thickness of unconsolidated sediments (depth to bedrock), is available in 1 km grid‐node spacing format for the Western CONUS and has been derived using various methods, including well depth, seismic reflection, and gravity (Shah & Boyd, 2018). Sediment thickness and land subsidence are not, however, used as predictors in our model but rather for comparison with the modelled withdrawals.

We obtain the in situ groundwater withdrawal data from ADWR (2020a) for the years 2002–2020. It is noteworthy that ADWR actively monitors pumping only within the AMA/INA region, and hence, withdrawals outside this area are unreported or unknown. As described previously, AMAs and INAs also have higher groundwater regulation. The data pre‐processing details are discussed in Section 2.3.1.

### 2.3 Methods

**FIGURE 2:**
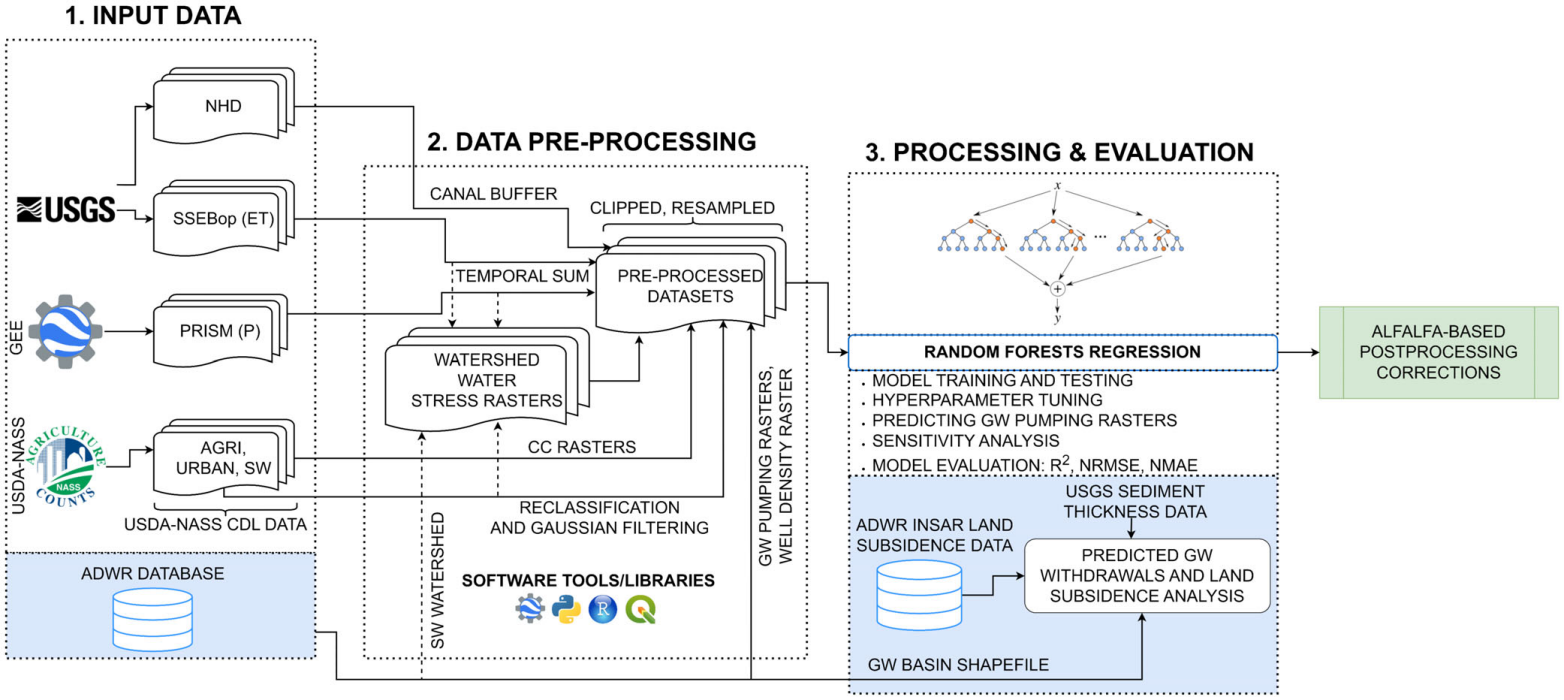
Major steps involved in the proposed workflow for estimating groundwater (GW) withdrawals in Arizona using open‐source or freely available tools, libraries, and data. The organization logos were obtained from the respective official websites, and the random forests figure was downloaded from the IU Digital Science Center (2013). The workflow figure has been reproduced from Majumdar et al. (2020) and modified accordingly. Here, the blocks represent the major steps in our workflow. Block 1 shows how the data are downloaded, after which, we use the preprocessing block to generate the predictor variables. This step includes statistical operations such as temporal sum, creating the groundwater pumping rasters, and other raster operations such as reprojection and resampling. The final block represents the machine learning steps such as model fitting and evaluation

The major steps involved in our workflow are conceptualized in Figure 2. We describe the data acquisition and pre‐processing steps in Section 2.3.1 and provide a brief review of our machine learning framework based on RF in Section 2.3.2.

#### 2.3.1 Data acquisition and pre‐processing

The data acquisition and pre‐processing workflow are similar to our previous work (Majumdar et al., 2020). We use the Google Earth Engine platform (Gorelick et al., 2017) to download the PRISM precipitation data for the growing season (January 1–December 31) of each year for the 2002–2020 time period. For the SSEBop ET and USDA‐NASS CDL data sets, we directly use the official data portals of USGS and USDA‐NASS, respectively. We temporally sum up the ET and P data for the growing season to obtain each year's cumulative ET and P. Next, we reclassified the CDL data into four classes depending on the pixel values representing agricultural (AGRI), urban (URBAN), surface water (SW), or other (OTHER) land use. Thereafter, we created three binary rasters corresponding to AGRI, URBAN, and SW and discarded OTHER. In order to introduce spatial context, we applied Gaussian filtering similar to Majumdar et al. (2020), which is further discussed in Section 3.1. Finally, we compute the mean land use densities between 2008 and 2020 and use them as predictors.

The watershed water stress rasters (WS_PA_ and WS_PA/EA_) were computed using ET, *P*, AGRI, URBAN, and the ADWR surface watershed shapefile following Equations (1) and (2). The CC raster is produced from the CDL data, wherein the pixel values are obtained by matching the crop names (from the CDL data) with the mid‐seasonal crop‐coefficient look‐up table provided by Allen et al. (1998). The well density raster (WD) is computed using the ADWR GWSI well location data across Arizona, wherein each pixel value indicates the number of wells within the pixel. As for the in situ groundwater (GW) withdrawals, the ADWR point data available over the AMA/INA region from 2002 to 2020 were rasterized, where each pixel value represents the cumulative withdrawals from all the wells within that 2 km × 2 km pixel area. In this context, since withdrawals outside the AMA/INA region are unknown, we included pixels where there were no wells implying zero withdrawals to increase the number of samples, thereby improving the model predictions. More specifically, we use the GWSI shapefile and the ADWR groundwater pumping CSVs to match the common wells (using well id) for the AMA/INA region. Areas outside AMA/INA where there are no wells, according to the GWSI data, have zero withdrawals, and these samples are included in the model training. However, if there is any well outside the AMA/INA region, that 2 km × 2 km pixel contains no data value as the pumpings are not reported.

A significant amount of surface water from the Colorado River is supplied for municipal and irrigation purposes to southern and central Arizona by the Central Arizona Project, or CAP (ADWR, 1983). Water rights, surface water availability, as well as water storage and re‐allocation, determine the spatio‐temporal variability in surface water deliveries from the CAP. While the detailed nuance of water allocation is beyond the scope of this study, we account for spatio‐temporal variability in surface water availability using canal data from the NHD and Colorado River discharge data. Our assumption is that, to first order, regions with inter‐connected canal systems that are linked to the CAP will have surface water deliveries that are a function of the Colorado River discharge. To this end, we identified features from the NHD that were coded as canals, created a 1 km buffer around each of these features, and dissolved them so that overlapping features were combined into one large buffered region. This resulted in a number of distinct regions with canals in many of the major irrigated areas in Arizona (Figure S1). Connected canal features that were linked to the CAP, as determined by both proximity and surface water allocations from ADWR (1983), were then rasterized with a value of 1, and all other cells were given a value of 0. Finally, annual discharge (AD) measurements from below the Parker Dam on the Colorado River were multiplied by this raster so that all cells in canal networks connected to the CAP were given a value representing the AD of the Colorado River. This AD raster was used as a predictor in our model, which could then learn a more nuanced relationship between proximity to canals, Colorado River discharge, and groundwater withdrawals.

All the raster data sets are clipped to the Arizona state boundary, reprojected to UTM 12N, and resampled using the nearest neighbour algorithm to 2 km resolution. We use the 2 km annual groundwater pumping rasters (created from the ADWR pumping CSV files) as reference rasters for reprojecting and resampling all the other rasters to 2 km resolution. This also includes the land use data. Essentially, all the predictors are resampled to 2 km resolution.

The entire workflow is fully automated and uses open‐source or freely available programming languages, tools, and libraries. In this research, we use Python 3 (Van Rossum & Drake, 2009) as the main programming backend for data acquisition, pre‐processing, and implementing the machine learning model. R (Venables, Smith, & R Core Team, 2021) and QGIS (QGIS Project, 2021) are used for statistical analysis and visualization purposes, respectively. The primary Python libraries used in our workflow include NumPy (Harris et al., 2020), SciPy (Virtanen et al., 2020), GDAL/OGR (GDAL/OGR contributors, 2021), scikit‐learn (Buitinck et al., 2013; Pedregosa et al., 2011), Rasterio (Gillies, 2013), GeoPandas (GeoPandas developers, 2021), and Pandas (McKinney, 2010).

Groundwater overdraft‐induced land subsidence has been widely reported across Southern and South‐Central Arizona since the 1940s (Conway, 2016). Groundwater level declines in unconsolidated aquifers in these regions containing geomechanically weak clay layers act as the primary driver of land subsidence. Subsidence can be monitored with InSAR. InSAR tracks the change in phase over time at microwave wavelengths (mm to 1 m). The change in phase can be related to ground deformation with cm‐scale accuracy or better and a spatial resolution of roughly 100 m. Primary sources of noise in InSAR data include vegetation growth and tropospheric effects. However, modern InSAR processing methods are capable of reducing noise by identifying stable pixels with limited vegetation growth and stacking multiple over‐lapping time frames to reduce tropospheric noise (Moreira et al., 2013). In arid regions such as Arizona, typical InSAR sources of noise are much smaller, and the data quality is high (Conway, 2016; Zebker et al., 1997). Extensive subsidence data have been processed using InSAR by ADWR (2021a).

#### 2.3.2 Machine learning with RF

RF (Breiman, 2001) is an ensemble machine learning algorithm that is extensively used in the remote sensing domain (Belgiu & Drăguţ, 2016). In this work, we use RF for addressing a multi‐variate regression problem where the objective is to accurately predict annual groundwater withdrawals given 10 different predictors (AGRI, URBAN, SW, CC, ET, *P*, WS_PA_, WS_PA/EA_, AD, and WD). We employed three splitting strategies for generating the training and test data based on holding out temporal, spatial, and spatiotemporal data (discussed in Section 3.1) to analyse the model performance.

For model evaluation, we relied on RF feature importance and different error metrics such as the coefficient of determination ($R^{2}$), root mean square error (RMSE), mean absolute error (MAE), normalized RMSE (NRMSE), and normalized MAE (NMAE), wherein the normalization is carried out by dividing the RMSE and MAE with the mean of actual groundwater withdrawals. Like Majumdar et al. (2020), the feature importance or Gini importance is computed using the total decrease in node impurity (variance) averaged over all the trees (Breiman et al., 1984). We also perform residual diagnostics and normality checks to understand possible bias in our model residuals (Hastie et al., 2001).

As for hyperparameter tuning, we optimized three parameters, *n_estimators* (number of trees), *max_features* (maximum number of features or predictors split during model training), *max_depth* (maximum tree depth), and *min_samples_leaf* (minimum number of samples required to be at a leaf node) using the scikit‐learn API (Buitinck et al., 2013; Pedregosa et al., 2011). We observed that *n_estimators* = 100, *max_features* = 5, *max_depth* = 18, and *min_samples_leaf* = 3 provided the best results (lower NMAE and NRMSE, and higher $R^{2}$) with all the other hyperparameters set to scikit‐learn defaults (scikit‐learn developers, 2021).

#### 2.3.3 Correcting for changes in alfalfa

**FIGURE 3:**
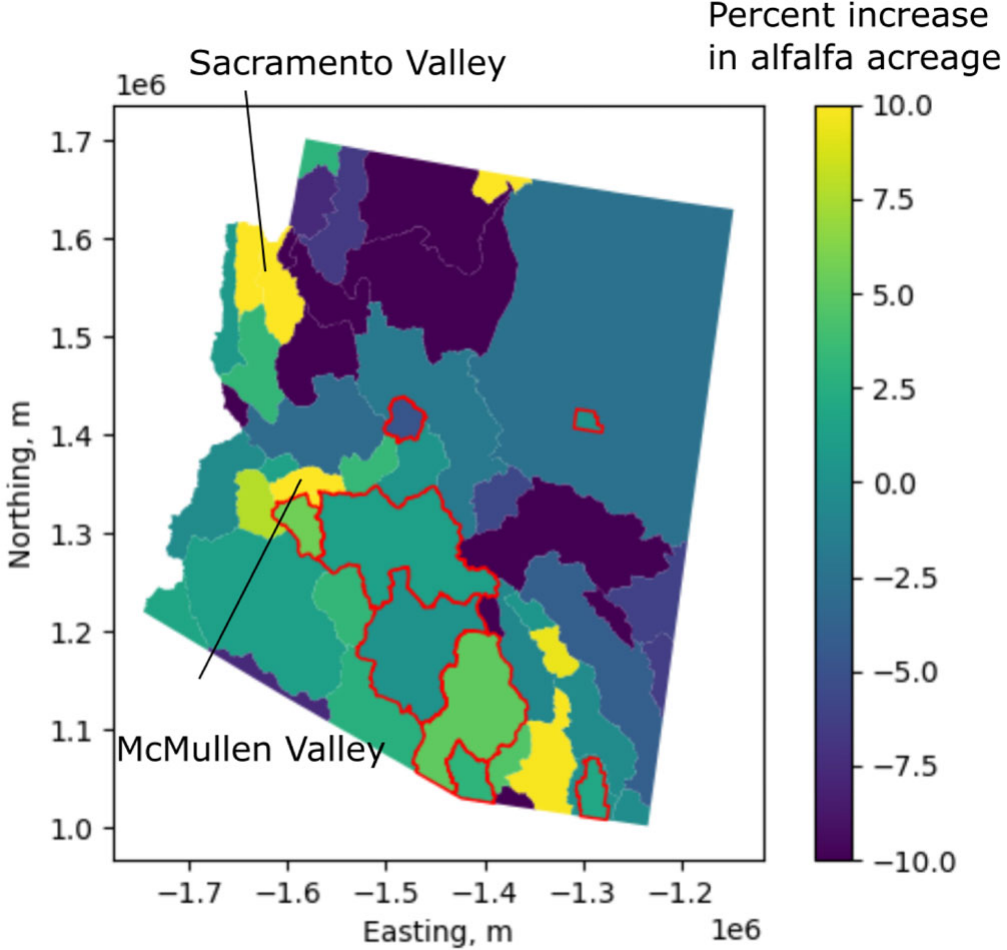
Average annual percent change in cultivated alfalfa area by groundwater basin. AMA and INA basins are shown with a red outline

Alfalfa is the most common crop grown in Arizona and also the most water‐intensive, with an estimated water use of 1646 mm compared to the average Arizona crop's estimated water use of 1280 mm (Frisvold, 2015). From 2008 to 2020, many unregulated groundwater basins in Arizona increased the amount of alfalfa grown. However, most INAs and AMAs in Arizona experienced little change in alfalfa, and none experienced the dramatic changes that occurred in basins such as McMullen and Sacramento Valleys (Figure 3).

This discrepancy is likely a function of regulation, as the more regulated INAs and AMAs are less likely to increase the planting of water‐intensive crops. Because our training data is located exclusively in INAs and AMAs, our model would not be able to identify the full impact of increasing alfalfa acreage. For this reason, we applied a post‐processing correction for regions that had changes in alfalfa acreage. To apply this post‐processing correction, we first calculated the fraction of all crops grown in AMAs and INAs during the training period that was alfalfa, $Fr_{\text{alfalfa}}=0.44$. Next, we computed the expected average water use over these regions given this fraction alfalfa, water use for alfalfa, and the average crop water use from Frisvold (2015), using Equation (3):

(3) $$\mathrm{WU}=Fr_{\text{alfalfa}}\times WU_{\text{alfalfa}}+\left(1-Fr_{\text{alfalfa}}\right)\times WU_{\text{average}}$$

$WU_{\text{alfalfa}}$ and $WU_{\text{average}}$ were determined from the values reported by Frisvold (2015) and referenced earlier. The average water use over the AMAs/INAs was computed using $Fr_{\text{alfalfa}}=0.44$, to be 1440 mm and served as our baseline. Next, we computed $Fr_{\text{alfalfa}}$ over each individual basin in Arizona, for all years from 2008 to 2020, and used Equation (3) to estimate water use for each basin and each year, $WU_{\text{basin year}}$. The correction factor was then determined by subtracting our baseline water use from the estimated water use, $WU_{\text{basin year}}$. This correction factor was then added to all pixels with at least 100 mm of water use to correct for any changes in alfalfa grown during the study period. This correction was only applied to regions outside of the training areas. We note that from 2002 to 2007, no statewide estimates of alfalfa were available at the resolution necessary, so we used the 2008 estimated alfalfa fractions for those years. To illustrate the method, a map of correction factors for 2020 is shown, as well as a time series of correction factors for the Harquahala INA and McMullen Valley in Figures S2 and S3, respectively.

## 3 RESULTS AND ANALYSIS

As indicated above, we used three data splitting strategies which involved leaving out temporal, spatial, and spatiotemporal data in succession in the analyses. For temporal data splitting, we chose 2002–2009 as the training data and 2010–2020 as the test data. In the case of spatial data splitting, we select the Harquahala INA for model validation as it has the largest spatial extent among the INAs, with an area of around 1983 km^2^ (Towne, 2014). This is a sufficiently large region to test the model but not so large that it reduces a substantial portion of the training data needed to produce a robust calibration. In areas outside the Harquahala INA, all the temporal samples (2002–2020) are used to train the model. The spatio‐temporal data splitting strategy is an amalgamation of the spatial and temporal data splitting workflows where we leave out Harquahala INA for all the years (2002–2020), and for other regions, we choose only 2002–2009 data for model training.

At first, we showcase the sensitivity analysis results in Section 3.1. Thereafter, we present the model results and analysis in Section 3.2, wherein the optimized parameters obtained from the sensitivity analyses are used.

### 3.1 Sensitivity analysis

**FIGURE 4:**
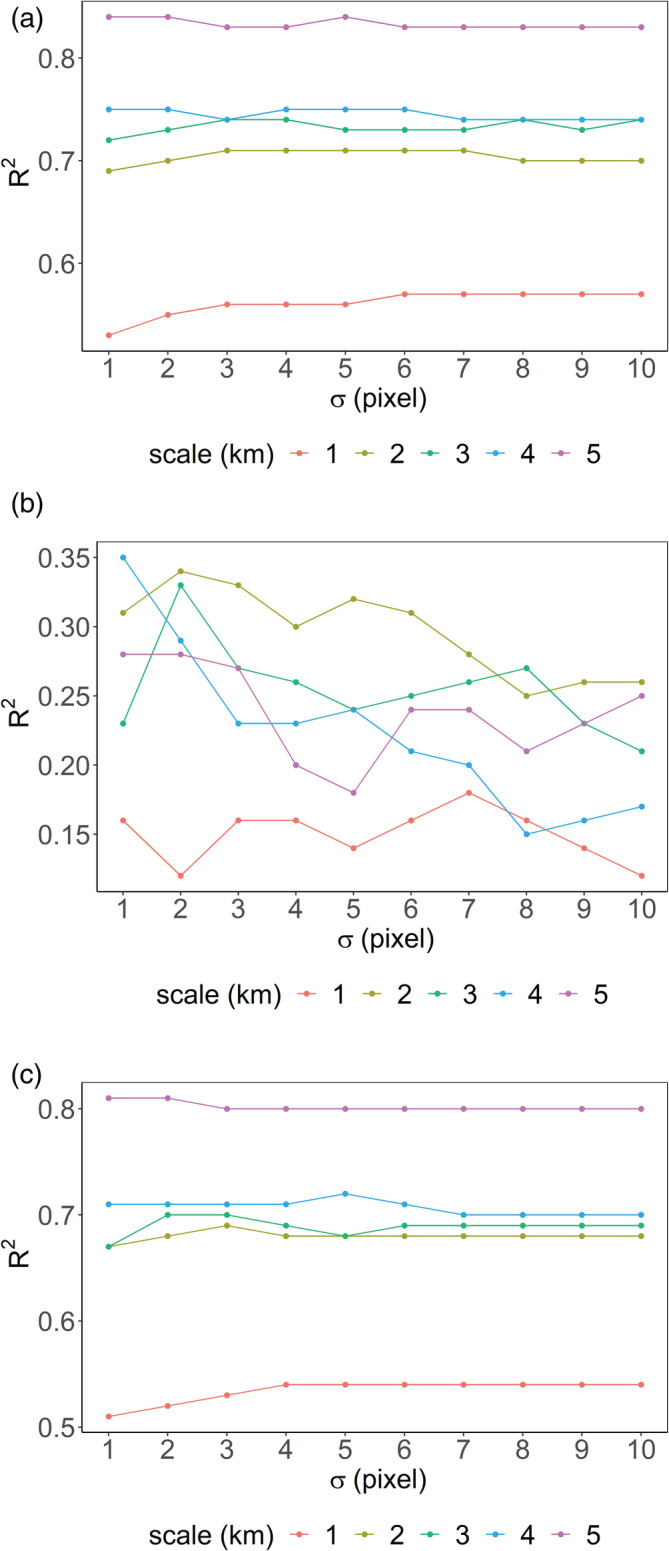
Sensitivity analysis for (a) temporal, (b) spatial, and (c) spatio‐temporal data‐splitting strategies

Two critical parameters that govern the way we use the remote sensing products in our proposed approach are the target scale of the estimates and the SD (σ) of the Gaussian kernel. For performing sensitivity analysis, we used the same RF hyperparameters as mentioned in Section 2.3.2 and varied the scale (in km) and σ (in pixels) between [1, 5] and [1, 10], respectively. In Figure 4, we compare the $R^{2}$ computed with varying σ and scale using the test data for the three data‐splitting strategies.

Since our model is re‐calibrated for each of these parameter combinations, the importance of various input features changes for each iteration. We find that varying scale and σ affect the performance on temporal, spatial, and spatio‐temporal validation datasets in different ways. Due to the coarser resolution of the PRISM product (originally at 4 km), precipitation, a key temporal predictor, has low feature importance (less variation with groundwater pumping) at finer scales. When we try to upsample a coarser grid to a finer one, we copy the same pixel value to those finer cells (nearest neighbour). So, there is less variation of the coarser products at finer scales, which effectively reduces the feature importance, thus reducing performance at overly fine scales. However, if we choose a higher scale (e.g., 5 km) by observing the trend that the model performs better as we increase the scale (Figure 4a,c), then we tend to lose out land‐use information (such as AGRI and URBAN), which is essential for extending the results over the entire state and for the subsequent land subsidence analysis (more on this is provided in Section 4). In addition, finer scales are better able to preserve the resolution of administrative boundaries, which likely have a strong effect on regulation and surface water availability. Note that we have a higher $R^{2}$ variation in Figure 4b because of the lesser number of validation samples (restricted to the Harquahala region only) as compared to Figure 4a,c.

When downsampling the high‐resolution CDL data to a coarser grid, the nearest neighbour algorithm assigns a zero AGRI or URBAN class value to the coarser cell with more non‐AGRI non‐URBAN classes. If there are smaller AGRI or URBAN regions within the coarser cell, we will lose such information, and the model would not predict groundwater pumping in those regions. Thus, finding an appropriate scale that is fine enough to preserve land use data while coarse enough for the model to learn to use precipitation and other coarse temporal predictors, is critical. We observe that the test $R^{2}$ is consistently high when the target scale is 2 km and σ=4 pixels (i.e., a spatial window of 8 km × 8 km). As a result, we find the target resolution of 2 km to be the most appropriate for this study.

### 3.2 Groundwater withdrawal estimates

#### 3.2.1 Holding out temporal data

**FIGURE 5:**
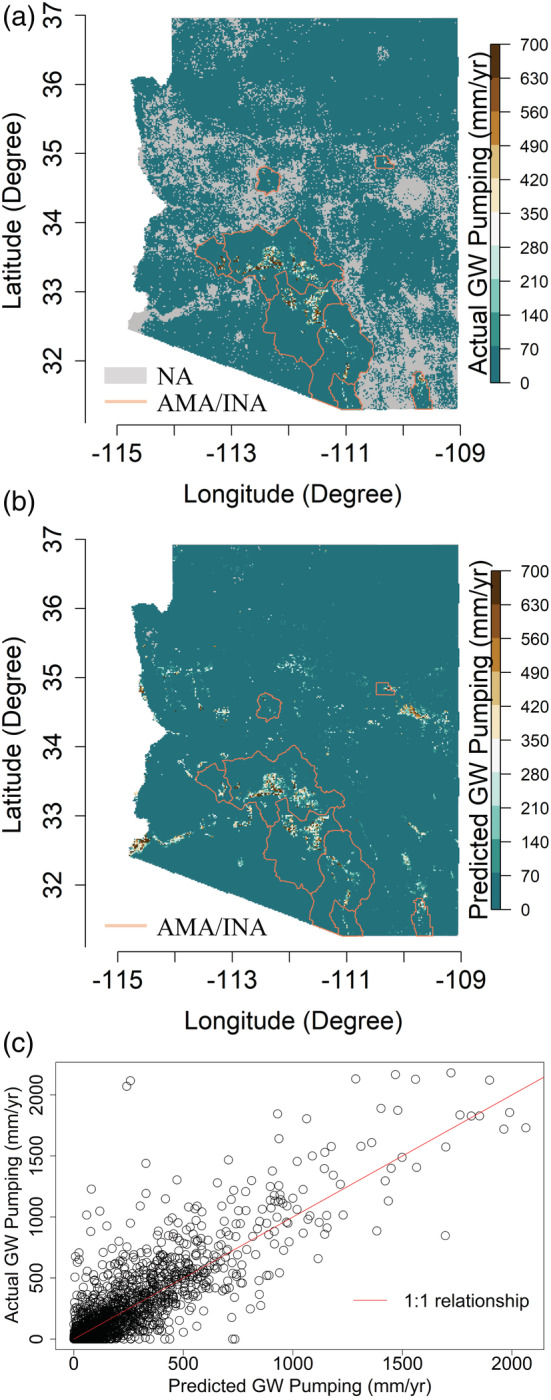
The mean of (a) actual groundwater (GW) pumping and (b) predicted GW pumping for 2010–2020, along with the (c) scatter plot of the actual and predicted values. In (a), the areas outside the AMA/INA region having zero withdrawals do not have any pumping wells as per the GWSI database

For the temporal data splitting strategy, the number of training and test samples are 471 978 and 648 945, respectively, with a split of 42%–58%. The mean actual and predicted groundwater withdrawals for the test years 2010–2020 are shown in Figure 5a,b, respectively. In Figure 5a, the grey pixels indicate areas where ADWR knows the well locations, but unlike the AMA/INA regions, the groundwater pumpings are not reported (denoted as NA). Thus, we do not use these pixels in the model training. However, we assume there is no pumping (or zero pumping) for any other pixel outside the AMA/INA region, which does not have a well location listed in the ADWR GWSI database. Thus, we include these ‘zero’ groundwater pumping pixels to increase the training and test data.

The training error metrics include $R^{2}\approx 0.9$, NRMSE≈1.31, and NMAE≈0.37 over the AMA/INA region, with corresponding test error metrics being $R^{2}\approx 0.7$, NRMSE≈2.34, and NMAE≈0.62, respectively. Moreover, we are able to extrapolate our estimates to the entire state of Arizona (Figure 5b), and these estimates are also constrained to the well locations meaning that if there are no wells, then no pumping is occurring. When we predict groundwater pumpings across the entire state of Arizona (Figure 5b), we only compute the error metrics for AMA/INA because we do not know the ground truth of the grey pixels in Figure 5a. However, we can extrapolate to other places because of the continuous nature of the predictor data sets.

**FIGURE 6:**
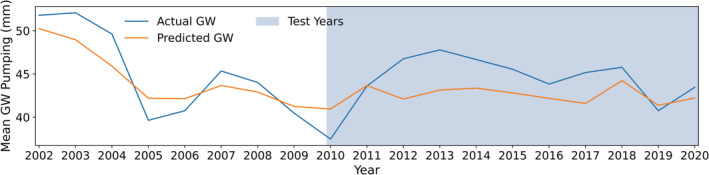
Mean actual and predicted groundwater (GW) pumping over the AMA/INA region for each year, with 2010–2020 being validation or test years

From the scatter plot in Figure 5c, we see a good fit between the actual and predicted pumping. The majority of the scatter points approximately follow the 1:1 relationship, even though there are some underpredictions and overpredictions. Additionally, the mean actual and predicted groundwater pumping for each year are depicted in Figure 6, showing that the model predictions for the test years (2010–2020) follow the actual ones reasonably well. More quantitative information about Figure 6 is shown in Table S1, where we provide the error metrics for the individual training and test years. Although there is some model misfit, the mean predictions for most validation years closely follow the actual mean pumpage. It is expected that training over a longer time period would allow the model to better capture spatio‐temporal relationships including the relationship of Colorado River discharge to pumping. Moreover, the location of the Colorado River gauging stations affects the overall trends generated from the model, for example, discharge data (https://sgp.fas.org/crs/misc/R45546.pdf) from the Lee's Ferry station in Arizona (Glen Canyon Dam, ~560 km upstream from the Parker Dam) improved the estimates for 2012 and 2013 but resulted in slightly higher misfits for 2015, 2016, and 2020 (Figure S4). In Figure 6, the mean predicted pumpings for 2016 and 2018–2020 are quite accurate, and our model is able to capture the temporal trends suitably. On the contrary, for the training years (2002–2009), we observe that the model does not capture the temporal trends. This could be attributed to the *max_depth and min_samples_leaf* hyperparameters, which control the model overfitting. Nevertheless, the satisfactory groundwater withdrawal predictions for the test or validation data highlight that our model has a good generalizing capability. Here, the feature importance (in decreasing order) corresponding to WD, AGRI, URBAN, SW, CC, AD, ET, WS_PA_, *P*, WS_PA/EA_ are 0.24, 0.15, 0.15, 0.12, 0.11, 0.08, 0.07, 0.04, 0.02, and 0.01, respectively (Figure S5).

**FIGURE 7:**
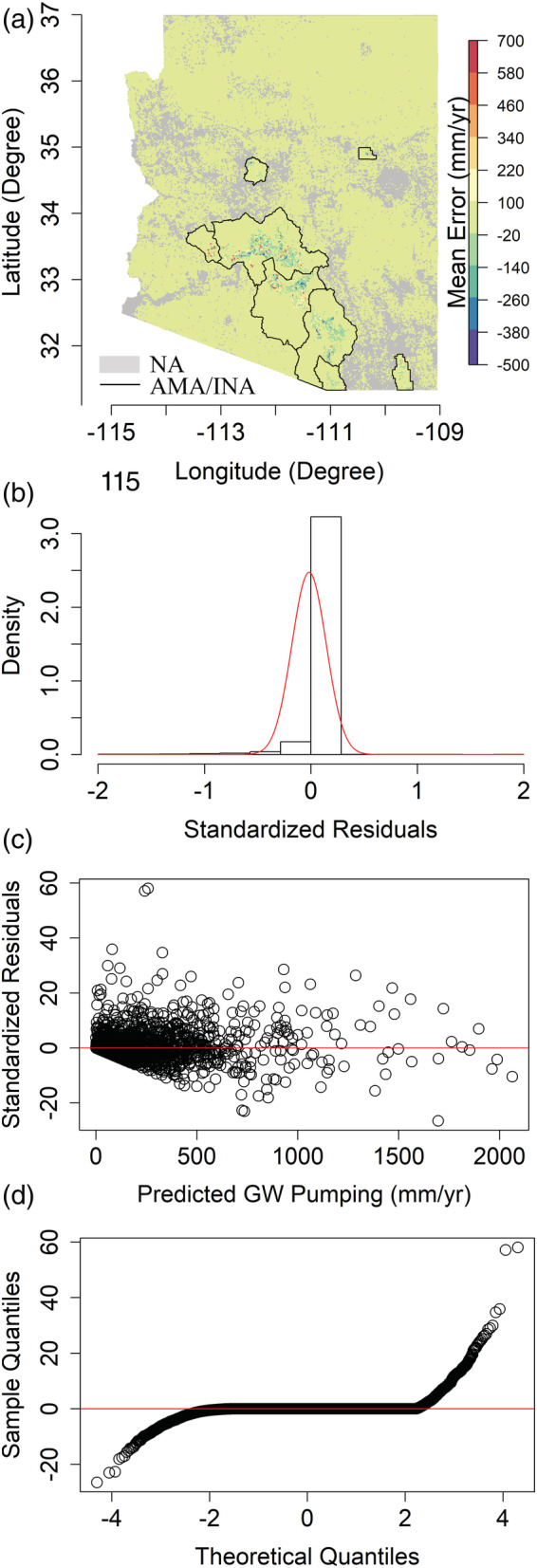
(a). Residual diagnostics (actual‐predicted) for the test data (2010–2020) showing (a) mean groundwater (GW) pumping error raster, (b) histogram showing the standardized residuals restricted within the [−2, 2] interval (the red line represents the Gaussian probability density function), (c) standardized residuals versus predicted pumping, and (d) Q‐Q plots. Note that the higher scatter in (c) as compared to (b) is because the standardized residuals are shown for the full range instead of constraining them to [−2, 2] like in (b)

Next, we show the residual analysis of the model estimates in Figure 7a–d. Figure 7a shows that in major pumping areas, the model underpredicts (negative Mean Error) more than it overpredicts, and most of these large errors are present in the Phoenix AMA; wherein there is extensive groundwater pumpage with annual cumulative withdrawal rates exceeding 2000 mm/year in some places. This observation is consistent with the RF prediction algorithm, wherein it tends to predict the mean of the training samples for unseen data (Breiman et al., 1984). The standardized residual histograms in Figures 7b and S6 show the residuals to be slightly skewed but closely follow a normal distribution, with the mean and SD of the error residuals being 0.08 mm/year and 31.99 mm/year, respectively. Similarly, Figure 7c shows no clear pattern in the residual scatter plot and that the residuals are clustered around 0. This implies that the residuals are independent and almost normally distributed, which is also depicted in Figure 7d. Even though there is a slight bias, 98.48% of the standardized residuals lie in the [−2, 2] interval, and thus, our model predictions are quite robust.

#### 3.2.2 Holding out spatial data

**FIGURE 8:**
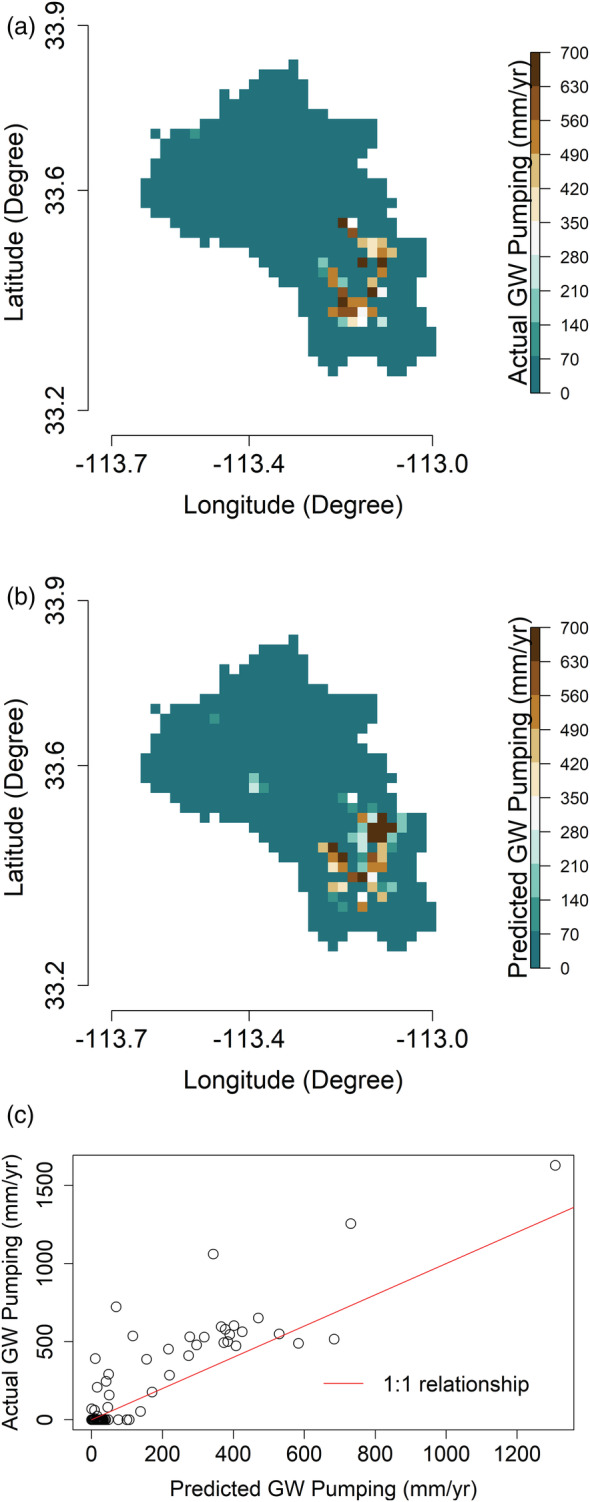
(a). The mean of (a) actual groundwater (GW) pumping and (b) predicted GW pumping over the Harquahala INA for 2002–2020, along with the (c) scatter plot of the actual and predicted values

Here, we show the model results obtained by holding out the Harquahala INA (2002–2020) from the training process. The total number of training and test samples are 1 111 585 and 9338, respectively. The mean actual and predicted groundwater withdrawals are shown in Figure 8a,b, along with the scatter plot in Figure 8c.

The training error metrics are $R^{2}\approx 0.91$, NRMSE≈3.11, NMAE≈0.34 (includes regions outside the AMA/INA also), respectively, with the corresponding test error metrics (only over the Harquahala INA) being $R^{2}\approx 0.43$, NRMSE≈3.56, NMAE≈0.93. Although the predicted pumpings are not as accurate as in Section 3.2.1, it is noteworthy that the model underpredicts or overpredicts for specific pixels and that for the entire Harquahala INA, the model accuracy is relatively high, with the mean actual and predicted groundwater withdrawals being 33.7 and 38.814 mm/year, respectively. As a result, our model would perform satisfactorily if we downsample these predictions to a coarser resolution, for example, at the INA scale. The error metrics are likely worse as the model predictors are mainly based on water demand (i.e., land use type and ET), but water could be pumped from a neighbouring pixel to supply water to a field that has a high demand. Because of this, visual inspection of Figure 8a,b shows good spatial agreement, even though the error metrics are worse than in the other test cases.

**FIGURE 9:**
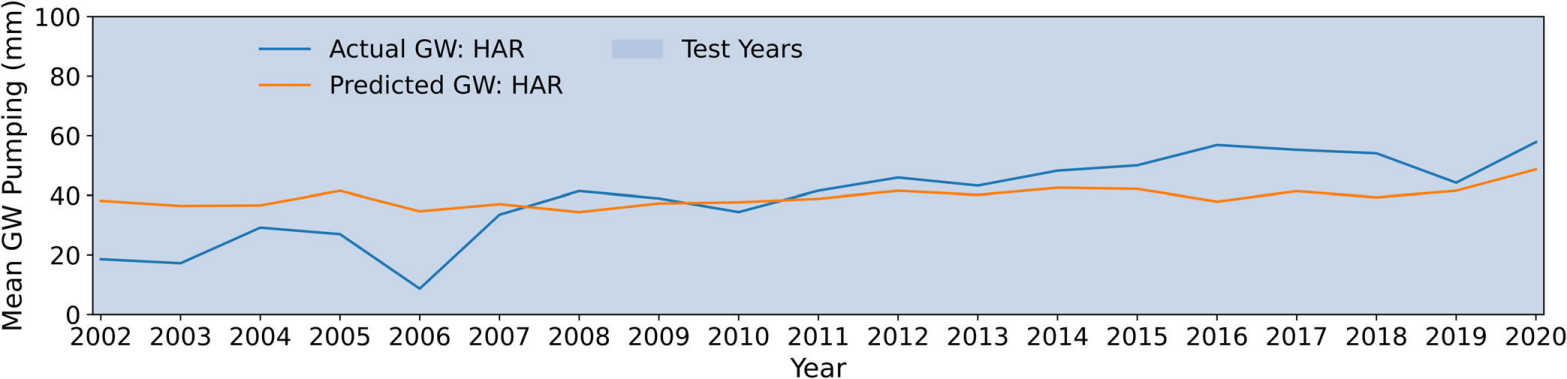
Mean actual and predicted groundwater (GW) pumping over the Harquahala INA for each year. It should be noted that this entire region was excluded from model training

In Figure 9, we observe the mean actual and predicted groundwater withdrawals over the Harquahala INA for each year from 2002 to 2020. Prior to 2008, our model over‐predicts withdrawals, but after 2008 it captures the magnitude and overall trend with reasonable accuracy. Since 2008, the acreage of alfalfa, which is the principal irrigated crop in the Harquahala INA, has roughly doubled (Figure S7). While we correct for changes in alfalfa acreage starting in 2008, we have no data on land use prior to 2008, so we assume 2008 values from 2002 to 2008. Due to the observed trend, it is likely that prior to 2008, alfalfa acreage was even lower, which would explain the discrepancy between the observed and predicted values.

**FIGURE 10:**
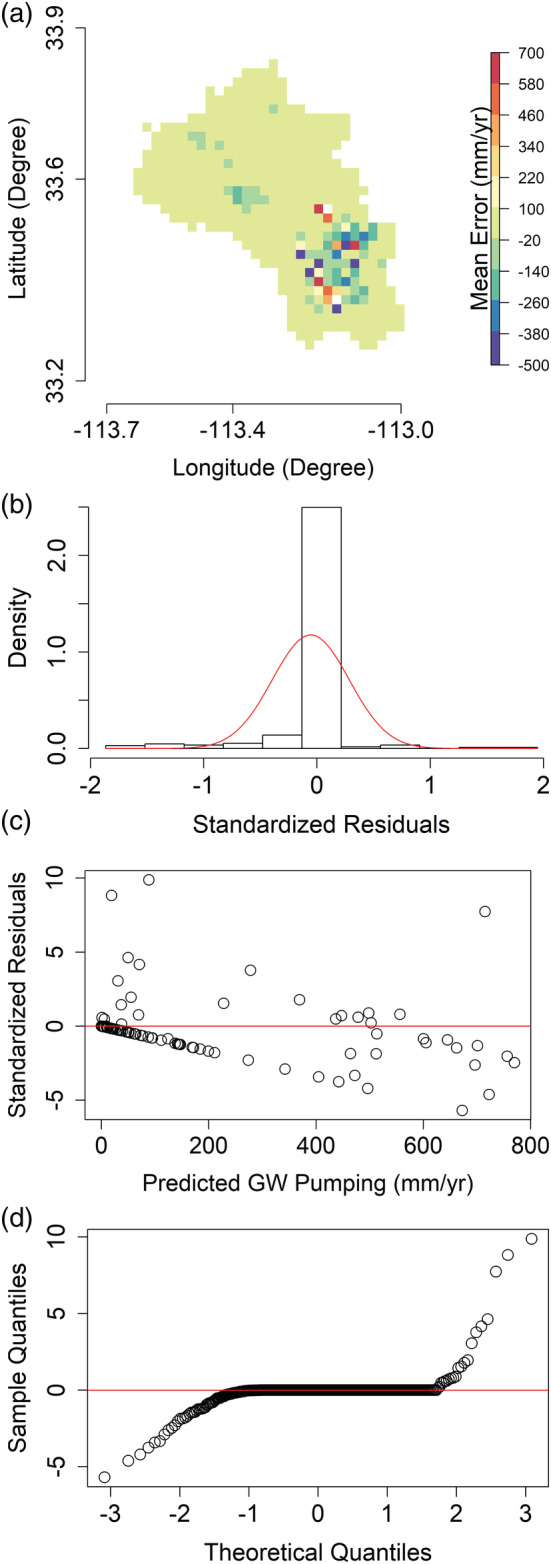
(a). Residual diagnostics (actual‐predicted) of the model predictions over the Harquahala INA with (a)–(d) representing similar figures as Figure 7

Next, we carry out residual analyses similar to Section 3.2.1, depicted in Figure 10a–d. Figure 10a shows that model predictions exhibit high pixelwise errors for areas with high pumpage but do quite well outside these regions. These pixelwise errors are because of the low generalizability of the model when we consider the spatial data splitting strategy. Since the model has only been trained from the areas outside the Harquahala INA, it tends to predict an average estimate of the training samples, an inherent property of the RF algorithm (Breiman, 2001). This is determined when comparing the mean actual and predicted withdrawals over Harquahala, which differ by 5.11 mm/year. Thus, our model tends to produce sufficiently accurate results over the entire Harquahala INA. However, we have high errors at the pixel level (2 km resolution) because of the low model generalizability. Nevertheless, 96.35% of the standardized residuals shown in Figure 10b–d lie in the [−2, 2] interval suggesting that the distribution of the residuals is approximately normal.

#### 3.2.3 Holding out spatiotemporal data

In this data splitting strategy, we have 468 044 training samples and 652 879 test samples, respectively, where 9338 samples in the test data belong to the Harquahala INA (2002–2020); the remaining test samples are drawn from other regions for the years 2010–2020. The train error metrics for the AMA/INA region are $R^{2}\approx 0.88$, NRMSE≈1.43, NMAE≈0.39, with the test error metrics being are $R^{2}\approx 0.69$, NRMSE≈2.38, NMAE≈0.63. We observe that these metrics closely follow those for the temporal data‐splitting strategy in Section 3.2.1, and the residual analyses are also similar. This similarity is also reflected in the feature importance of 0.25, 0.15, 0.15, 0.12, 0.12, 0.08, 0.07, 0.04, 0.02, and 0.01 (WD, AGRI, URBAN, CC, SW, AD, ET, WS_PA_, *P*, WS_PA/EA_) which are highlighted in Figure S5. The test error metrics corresponding to the AMA/INA regions are summarized in Table 1, which suggests that leaving out temporal samples slightly improves the results over the Harquahala INA.

**TABLE 1:** Test error metrics obtained over the AMA/INA regions (listed by full name and ADWR AMA/INA acronym)

AMA/INA	Test error metric		
	$R^{2}$	NRMSE	NMAE
Phoenix (PHX)	0.69	2.06	0.61
Pinal (PIN)	0.76	1.86	0.51
Tucson (TUC)	0.51	4.1	0.97
Santa Cruz (SCA)	0.65	2.87	0.76
Prescott (PRE)	0.55	3.58	1.05
Harquahala (HAR)	0.45	3.286	0.81
Douglas (DIN)	0.57	1.9	0.72
Joseph City (JCI)	0.73	2.43	0.83

*Note*: Here, the Harquahala INA is left out from model training whereas, for the other regions, 2010–2020 are used for testing. The color signifies that out of all the AMA/INA regions, only the Harquahala INA is left out from the model training.

For the Harquahala INA, 96.35% of the standardized residuals lie within [−2, 2], and the error metrics are better than those in Section 3.2.2. Also, 98.48% of the standardized residuals obtained for the entire test data belong to [−2, 2], and hence, the residuals approximately follow a normal distribution.

#### 3.2.4 Model validation outside of AMAs and INAs

As stated in Section 2.3.3, the AMAs and INAs, which are used to train our model, regulate groundwater use, while regions outside of the AMAs and INAs have little groundwater regulation. For this reason, our model may under‐predict groundwater use outside of AMAs and INAs, if those regions use more water under similar climate, geologic, and crop use scenarios. Here we compare our model estimates with two observations that are a consequence of groundwater withdrawals: declining groundwater levels and subsidence. While this is not a direct validation of our model, it does allow us to evaluate if there are systematic biases in our modelling approach due to the nature of available data for training our model.

##### Comparison with groundwater levels

To test to what extent our model may under‐predict withdrawals in unmanaged basins of Arizona, we compared our model‐derived estimates of groundwater use from 2009 to 2019 with groundwater level changes over the same period obtained from ADWR (2021b). While aquifer storativity and recharge, which govern the extent to which withdrawals will cause declines in groundwater levels, vary from basin to basin, we expect that if our withdrawal estimates are not strongly biased, the relationship between withdrawals and groundwater level changes (i.e., the slope) will have a similar distribution for both unmanaged and AMA/INA basins. We computed a best‐fit line with the least‐squares method for both AMA/INA basins and unmanaged basins. Groundwater basins containing underground storage facilities or groundwater savings facilities, which are both used in the state of Arizona to recharge aquifers (https://waterbank.az.gov/storage-facilities), were excluded from our comparison, as recharge rates are artificially high and reduce the impact of groundwater withdrawals on the head.

**FIGURE 11:**
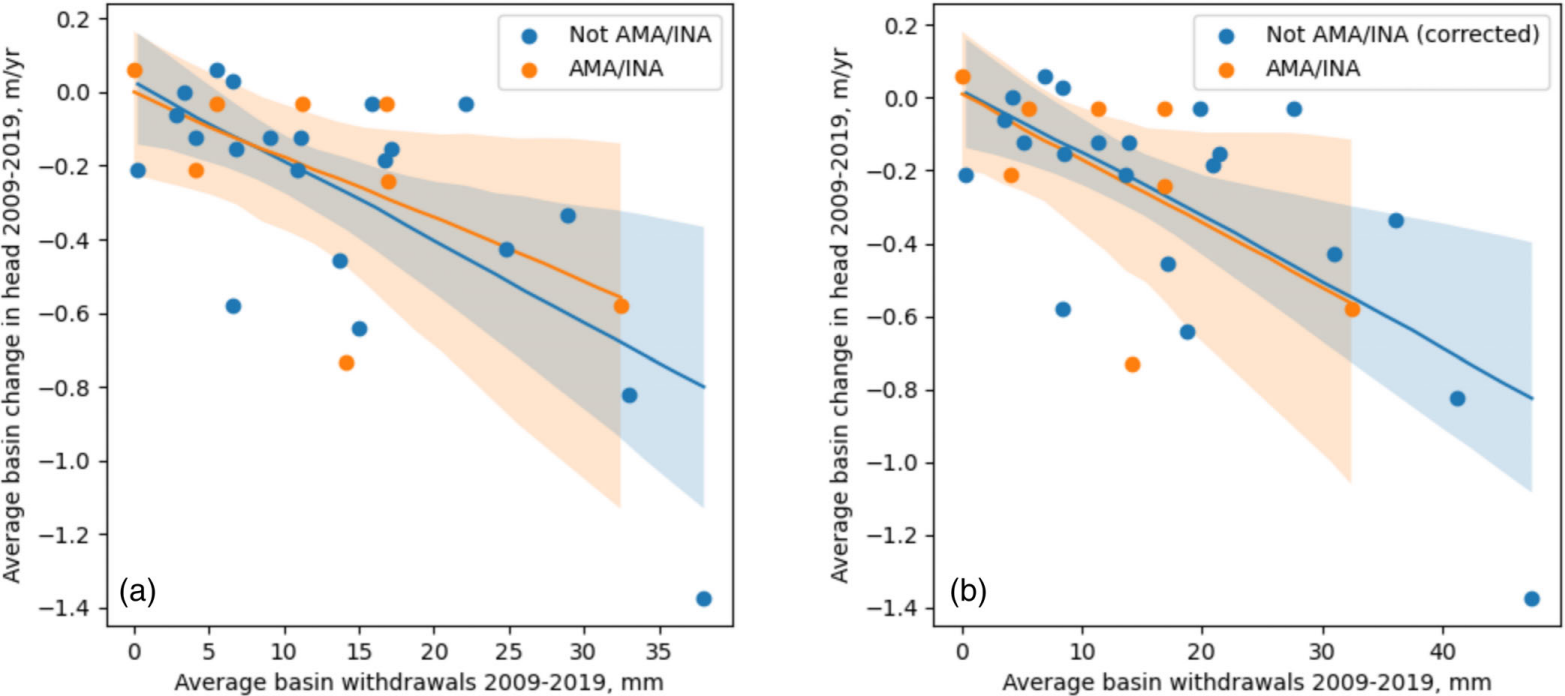
(a) Average basin change in head from 2009 to 2019 (ADWR, 2021b) compared with average basin withdrawals over the same time period, estimated from our model. Solid and shaded areas represent best‐fit and 5th to 95th percentile confidence intervals from bootstrapping, respectively. (b) Same as (a), but with a 25% increase in pumping in unmanaged (not AMA/INA) basins

The results of this analysis are shown in Figure 11. To determine the confidence interval of these best‐fit lines, we performed a bootstrapping analysis, in which we randomly sampled 60% of the value pairs and computed the slope and intercept of the best‐fit line 500 times. We then determined the 5th and 95th percentiles of the resulting datasets. These confidence intervals are shown as shaded regions in Figure 11a.

It is clear from the results in Figure 11a that, on average, there is a steeper drop in the head for an equivalent volume of groundwater withdrawals in unmanaged basins than in AMA/INA basins. While some scatter is likely explained by differences in recharge and storativity, and the confidence intervals are overlapping, these results suggest that our model may under‐estimate withdrawals in unmanaged basins. Higher withdrawal rates in unmanaged basins are plausible and have been suggested by ADWR (2018a), who found in groundwater modelling of the Willcox Basin that higher‐than‐expected pumping rates were required to match observed head declines.

If we increase our predicted withdrawals by 25% in unmanaged basins (Figure 11b), the slopes match, with a z‐score of 0.9, indicating there is no statistically significant difference between the distribution of slope values from the bootstrapping analysis. While these results support the hypothesis that more pumping occurs in unmanaged basins, further analysis would be required to determine exactly how much more is occurring, specifically accounting for variability in aquifer type and recharge.

##### Comparison with land subsidence

As discussed in Section 2.3.1, extensive subsidence due to groundwater withdrawals in Arizona has been mapped by ADWR (2021a). The presence and amount of clay, as well as the magnitude of groundwater level decline, are the two primary drivers of land subsidence. Subsidence is also a function of aquifer confinement, as confined aquifers experience a larger drop in water level relative to the change in storage than unconfined aquifers, and deformation is the principal mode of storage loss in unconsolidated, confined aquifers (Smith & Majumdar, 2020). Because of the many factors affecting land subsidence, the relationship between groundwater withdrawals and subsidence is non‐linear and complex (Smith & Knight, 2019). For this reason, we present here a qualitative comparison of our pumping estimates and land subsidence in confined aquifers, where subsidence due to groundwater pumping is most prone to occur due to the hydrogeologic conditions. We did not use subsidence as a model predictor so we could validate the spatial patterns of withdrawals produced by our model in regions prone to subsidence, and evaluate aquifer properties related to subsidence.

**FIGURE 12:**
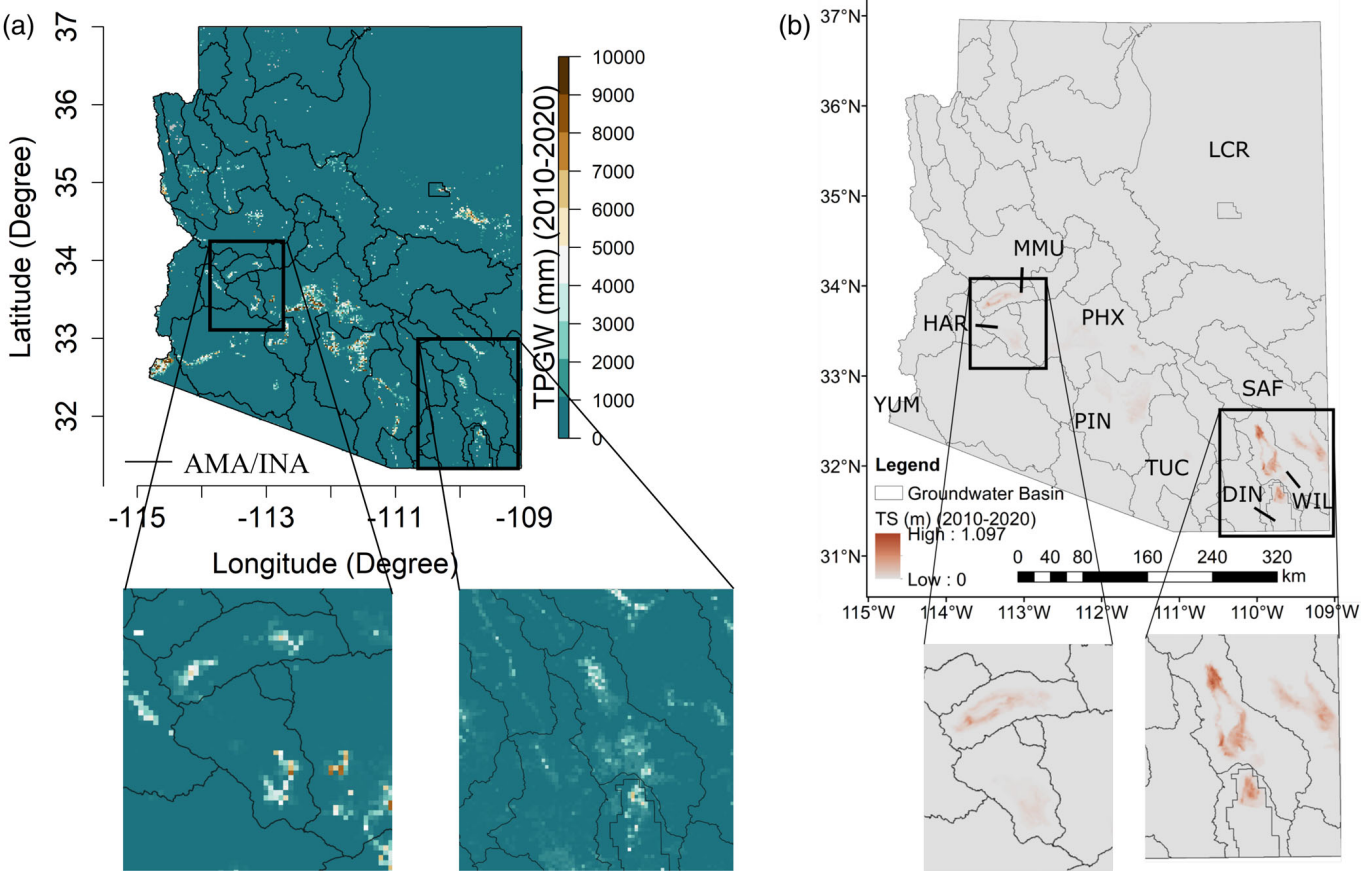
Maps showing (a) modelled groundwater withdrawals, and (b) subsidence observed with InSAR at 2 km spatial resolution

Aquifer confinement is shown in Table 2. Basins in the southeast portion of Arizona (SAF, WIL, and DIN) and some parts of west‐central Arizona (MMU) contain either semi‐confined or confined aquifers. In addition, HAR contains an unconfined and partially confined aquifer. These basins are highlighted in Figure 12a,b. There is a good spatial agreement between our modelled groundwater withdrawals and observed subsidence in these basins, both within AMA/INA basins (DIN) and unmanaged basins (SAF, WIL, and MMU). HAR, which contains both unconfined and partially confined aquifers, has the smallest subsidence signal, which is expected because it is the least confined. We do not quantify the correlation of these variables because the rate of subsidence is also driven by clay content and spatial patterns of groundwater decline, which have a complex relationship to withdrawals. However, the visual agreement between subsidence and withdrawals in confined basins outside the AMA/INA basins serves as a first‐order validation of our method's ability to estimate withdrawals in regions with no training data.

**TABLE 2:** The mean sediment thickness (rounded to two decimal places, Figure S8) and the TS/TPGW ratio (rounded to five decimal places) over each groundwater (GW) basin experiencing land subsidence

GW Basin	Mean sediment thickness (m)	TS/TPGW	Confinement of principal aquifer	Reference
RAN	197.77	0.00003	Unconfined	Tillman et al. (2011)
MMU	325.96	0.1065	Confined	Stolley et al. (2020)
HAR	361.45	0.03082	Unconfined/partially confined	Stolley et al. (2020)
PHX	290.66	0.00399	Unconfined	M. M. Miller and Shirzaei (2015)
PIN	339.97	0.01217	Unconfined	Rascona (2006)
TUC	260.53	0.00099	Unconfined	Eastoe and Gu (2016)
DOU	12.07	0.00461	Unconfined^a^	Coates and Cushman (1955)
DIN	324.58	0.09838	Partially confined	Coates and Cushman (1955)
WIL	240.79	0.15264	Partially confined^b^	ADWR (2018a)
SAF	207.95	0.10936	Partially confined	Corkhill (2015)

*Note*: The color signifies regions with high TS/TPGW ratio (> ~10%).

^a^ Wells along the edge of the basin, where this region lies, were interpreted by Coates and Cushman (1955) not to have confining layers.

^b^ Most wells drilled in the area that is presently subsiding at the time Coates and Cushman (1955) was produced were shallow (average depth of 89 m), while wells drilled after 1955 were much deeper (average depth of 145 m, ADWR (2018a)). Coates and Cushman (1955) reported mostly unconfined conditions with confining conditions observed at many deep wells (100 m depth and greater). For this reason, we consider the main aquifer at present to be partially confined.

## 4 DISCUSSION

The modelling results and analyses highlight that the best error metrics are obtained using the temporal data‐splitting strategy, similar to our earlier findings for Kansas (Majumdar et al., 2020). In this research, we observe that the spatially static land‐use predictors (particularly WD, AGRI, and URBAN) receive higher importance than the spatio‐temporal predictors, ET, *P*, WS_PA_, and WS_PA/EA_. Even though the watershed water stress metrics (WS_PA_ and WS_PA/EA_) are considered less important, these act as additional proxies for surface water availability (Smith & Majumdar, 2020); thus, removing these predictors reduces the model performance.

The sensitivity analyses carried out in Section 3.1 provide additional insights into the choice of scale and σ. We see that for the temporal and spatio‐temporal data splitting strategies, the variation of the test $R^{2}$ with σ is low, and scale is primarily responsible for changing the model performance. This is because the high‐resolution predictors (AGRI, URBAN, SW) are smoothed out at higher scales, and the variability of the in situ groundwater withdrawals is also reduced. Since we have less variability of the predictor variables at higher scales (also fewer pixels), σ, which essentially is the window size in pixels, will not significantly affect the model performance. As a result, when we spatially extrapolate the model estimates to the entire state of Arizona at higher scales, we tend to not predict withdrawals in smaller regions exhibiting substantial agricultural activities where the actual pumpage is unknown or unreported (i.e., areas having NA values in Figure 5a). This is because agricultural land use is occurring at a much finer scale than the scale of the model. However, at lower scales (<3 km), the model performance drops because of the coarseness of the spatio‐temporal predictors, particularly precipitation (PRISM), and because variation in the groundwater withdrawals is smoother, thus more predictable, at coarser scales. Despite these challenges, high‐resolution predictions are crucial for improved groundwater management. Because of this and the consistently high metrics across our three validation approaches, we consider a 2 km scale to be the most appropriate for our model. This model allows us to extend the model predictions over Arizona (Figure 5b) and perform a visual analysis of the unreported areas lying outside the AMA/INA region. In addition, we can appropriately relate land subsidence to groundwater withdrawals, as discussed below.

### 4.1 Relating land subsidence to groundwater withdrawals

As noted in Section “Comparison with land subsidence” and Figure 12a,b, there is a good visual agreement between the estimated withdrawal and subsidence datasets in basins with semi‐confined and confined aquifers, but many unconfined aquifers with significant pumping in Figure 12a that show little or no subsidence in Figure 12b. Although unconfined aquifers can have significant subsidence if the withdrawals are high enough to cause extreme drops in water level, confined aquifers are much more prone to subsidence because the water level drop relative to withdrawals is much higher (Fetter, 2001). In PHX, TUC, and PIN, which are primarily unconfined aquifers, significant historical subsidence occurred due to extreme groundwater overdraft. Recently, managed aquifer recharge in those basins has reversed water level declines and greatly reduced subsidence, although there is still some residual subsidence from historical water level declines (M. M. Miller et al., 2017).

Groundwater withdrawals cause a loss of aquifer storage, but the amount of storage lost depends on the magnitude of recharge and groundwater inflow from neighbouring aquifers. Aquifer storage loss is usually a relatively small fraction of total withdrawals. While the relative portion of groundwater withdrawals that results in storage loss varies by region, Butler et al. (2016) showed that in the heavily irrigated High Plains Aquifer of Kansas, groundwater storage loss was, on average, 7%–22% of the total withdrawals.

In confined aquifers, subsidence represents a lower bound on the loss of aquifer storage (Smith et al., 2017) because the loss of confined aquifer storage is accommodated by either loss of pore space (i.e., compaction and subsidence) or expansion of pore‐water, the latter of which is often not significant in unconsolidated aquifers (Smith et al., 2017). For this reason, we can make first‐order estimates of aquifer storage loss in basins whose aquifers are primarily confined and compare these with our estimates of groundwater withdrawals.

**FIGURE 13:**
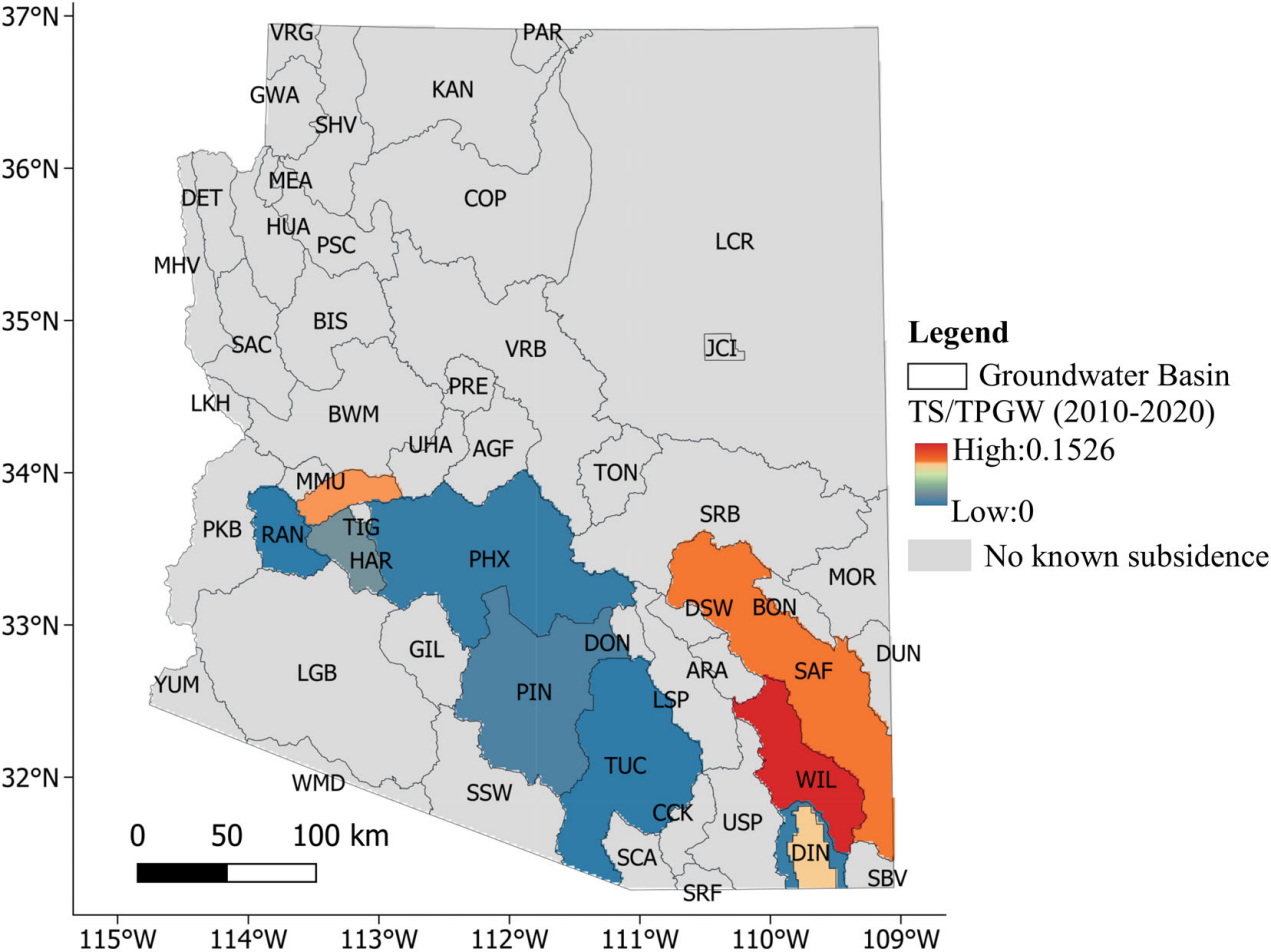
Total subsidence (TS) divided by the total predicted groundwater withdrawals (TPGW) estimated by our model. Confined or partially confined aquifers have average ratios of roughly ~0.1, or ~10%

We illustrate these principles in Figure 13, where we show the ratio of total subsidence to total withdrawals within each basin containing subsidence data. In confined aquifers, it is a first‐order estimate of the percentage of withdrawals that result in a loss in aquifer storage. Unconfined aquifers shown in Table 2 all have very low values, indicating little loss of storage due to compaction relative to pumping. The primary mechanism for storage loss in unconfined aquifers is the drainage of pores (Fetter, 2001), so this is expected. Conversely, confined aquifers in the Willcox (WIL), McMullen (MMU), Safford (SAF), and Douglas (DIN) basins all have high ratios, ranging from 0.1 to 0.15, or 10%–15%. These values are within the range of values estimated by Butler et al. (2016) in Kansas using a different method. The average ratio of subsidence to withdrawals in unconfined aquifers, excluding PIN, PHX, and TUC due to managed aquifer recharge, is ~0.002, while the average ratio of subsidence to withdrawals in confined aquifers is ~0.1. Due to the substantial difference in this ratio between aquifer types, it could also be used to identify the presence of confined or unconfined aquifers.

The ratio is also useful in estimating water budgets in confined aquifers. However, doing so relies on the assumption that withdrawal patterns have not dramatically changed over the past several decades, as residual compaction from previous withdrawals could also be affecting the subsidence signal (Galloway & Burbey, 2011), and some aquifer storage loss that has occurred may not have resulted in deformation yet. While these factors limit the interpretability of our results to some extent, we consider the apparent difference in ratios between confined and unconfined aquifers, as well as the similar relationship of storage loss to withdrawals we found in confined aquifers (~10%) compared with Butler et al. (2016) who found they ranged from 7% to 22% in Kansas, to suggest that this approach is useful for order of magnitude water budget analysis in confined aquifers.

### 4.2 Potential impact of pumping on surface water supply

**FIGURE 14:**
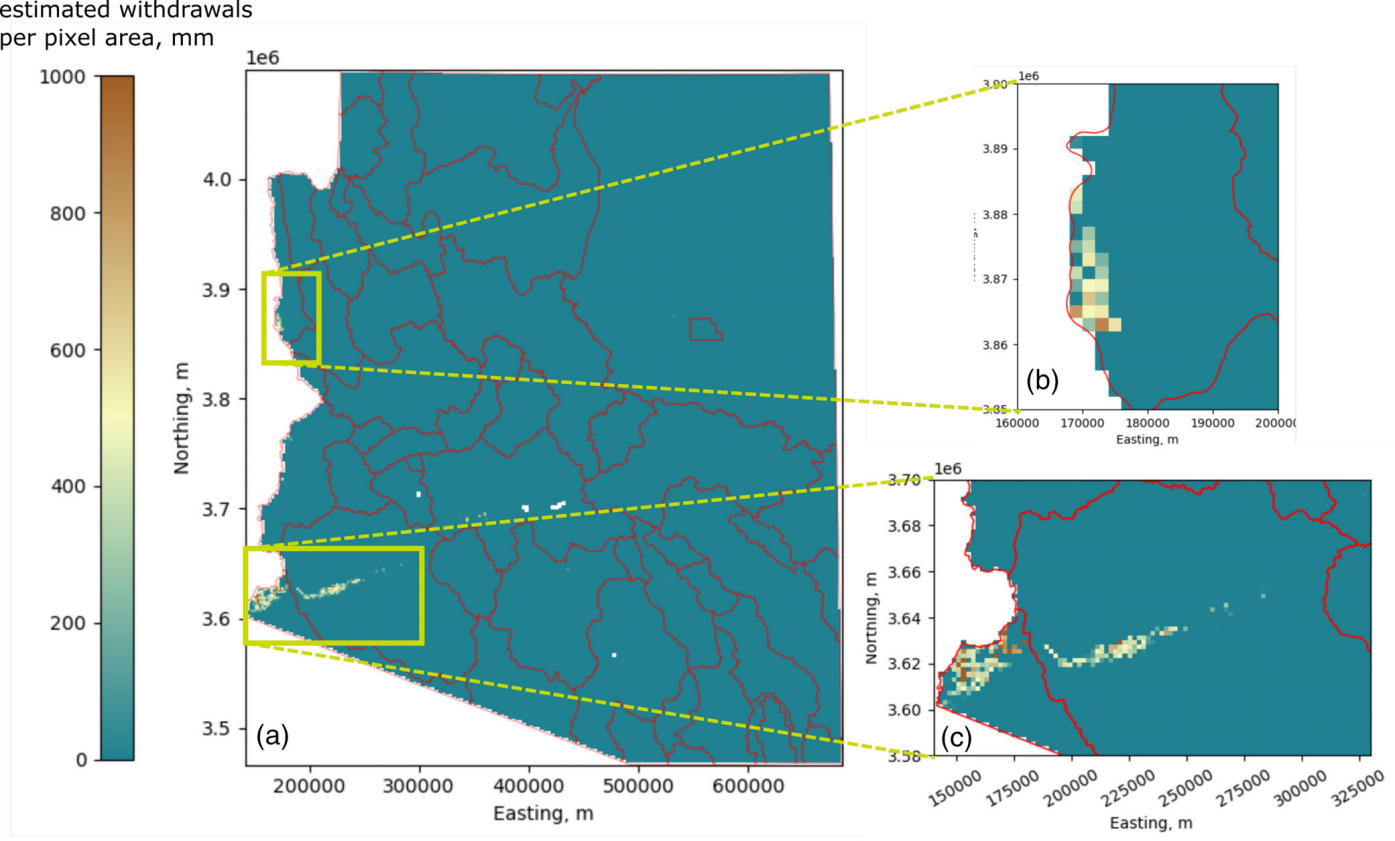
(a) Withdrawals, in mm per pixel area, that are considered to directly influence surface water supplies, with the most significant withdrawals occurring in (b) Lake Mohave Basin and (c) Yuma and Lower Gila basins

In some portions of Arizona, most notably the Yuma basin (southwest corner of Arizona), a significant amount of groundwater pumping occurs near the Colorado River (USBR, 2018). Pumping near a river, particularly when the water table is shallow and groundwater flows to the river, can have a significant impact on surface water supplies. To assess regions with groundwater withdrawals that potentially impact surface water supplies (Figure 14), we mapped floodplains in Arizona containing shallow wells. We mapped floodplains by first identifying stream reaches with catchment areas of at least 40 000 km^2^. The only reaches that met this criterion were the Colorado River and the Gila River, a tributary of the Colorado River with significant streamflow, although much of the water is used for irrigation upstream of the confluence with the Colorado River (USBR, 2018; USGS, 2022). We then identified all regions within 20 km of these reaches that had a grade (vertical relief divided by horizontal distance) of less than 0.4% and defined these as floodplains. Finally, we identified all pixels within our 2 km × 2 km resolution model within these floodplains with an average well depth of 50 m or less. Due to the presence of shallow wells indicating a shallow water table, and the proximity to surface water supplies, we consider these areas to have the highest risk of negatively influencing surface water supplies.

### 4.3 Uncertainty associated with the remote sensing products

Compared to our earlier work in Kansas (Majumdar et al., 2020), in this study, we selected the SSEBop ET product (Senay et al., 2013) instead of the MODIS (Moderate Resolution Imaging Spectroradiometer) Global Evapotranspiration Project (MOD16) data, because the latter has missing values over urban areas (Reitz et al., 2017). Hence, if we used MOD16, we would have left out several training samples predominantly from the Phoenix AMA. Regarding the uncertainty assessment of the SSEBop product, M. Chen et al. (2016) found that it performs satisfactorily in estimating ET with an $R^{2}\approx 0.86$ calculated against eddy covariance measurements at 42 AmeriFlux tower sites from 2001 to 2007. More specifically, $R^{2}\approx 0.92$ and RMSE≈13mm/month were obtained over croplands which suggests that the SSEBop product can be suitably used for this study. In addition, relative errors of less than 20% were observed across multiple AmeriFlux towers which further justifies the use of this product in our research.

The PRISM group (Daly et al., 2008) provides an extensive database of precipitation estimates using a network of weather stations. Although PRISM estimates have more errors in higher elevations (Henn et al., 2018), Stillman et al. (2016) found that for their study area in south‐eastern Arizona, PRISM provided the best correlation among the products they compared based on interannual timescales. Moreover, in the US corn belt, Mourtzinis et al. (2017) observed that the PRISM precipitation estimates lie within ±19% (RMSE) of the weather‐station measurements during the growing season. In addition, the higher spatial resolution of this product (4 km) is particularly suited to our study compared to globally available coarser (~10 km) products like the Global Precipitation Mission (GPM) data sets (Huffman et al., 2019). Thus, we consider the PRISM precipitation product to be an appropriate choice for our study, considering the robustness, availability, and maintainability of this data set.

In this research, we used the USDA‐NASS CDL land‐use products available over Arizona from 2008 to 2020 and computed the mean land use densities as specified in Sections 2.2 and 2.3. These CDL maps have sufficiently high crop classification accuracy, for example, the CDL 2015 data set has an overall crop classification accuracy of 89.6% for the entire state with 262 134 pixels accurately classified with a Kappa coefficient of ~0.87 (USDA‐NASS, 2015). Since USDA‐NASS CDL data are specifically tailored to the CONUS region at a sufficiently high spatial resolution (30 m), we considered this to be an appropriate product for our workflow as opposed to the globally available land cover products such as the MODIS land cover (Friedl & Sulla‐Menashe, 2019). However, we do note that CDL data are not available for the entire study period, yet there appear to be some systematic land use changes that affect our model accuracy. The static nature of this predictor thus does introduce some error to our model results.

Like Majumdar et al. (2020), the RF model can automatically learn from the consistent biases in these products. However, random errors, rather than systematic errors, are more likely to impact the model performance negatively.

## 5 CONCLUSIONS

In this research, we successfully advance our earlier work in Kansas (Majumdar et al., 2020) and extend it to the state of Arizona by providing new insights, particularly on model sensitivity. We also relate land subsidence to the predicted groundwater withdrawals and suitably demonstrate the extensibility of our approach at an even higher resolution (2 km vs. 5 km in our previous study), considering the fact that in situ pumping data are only available over the AMA/INA region.

Here, we develop an improved integrated workflow combining different openly available data sets (remote sensing, modelled, look‐up tables, and GIS‐based) into a RF‐driven machine learning framework and provide a thorough sensitivity analysis related to target scale and Gaussian filtering of the land‐use products. Moreover, we perform a more robust analysis by designing three different data‐splitting strategies (temporal, spatial, and spatio‐temporal) and observe that the temporal data‐splitting technique works best. Additionally, the RF feature importance and their relation to the spatial scales are discussed. We also developed a new approach that integrates InSAR and groundwater usage data to estimate loss of confined aquifer storage and improve the characterization of aquifer properties and conditions.

Even with the increasing global push towards sustainable groundwater management practices and water security in general, active monitoring of groundwater withdrawals is still limited to only a few regions worldwide. In this work, we successfully demonstrated the practicability and extensibility of our machine learning‐based approach, which could aid water managers in putting such water management efforts in traction.

## Supporting information


**FIGURE S1.** Canal buffer map showing features from the NHD that were coded as canals. Here, we created a 1 km buffer around each of these features, and dissolved them so that overlapping features were combined into one large buffered region.
**FIGURE S2.** Alfalfa‐based postprocessing correction factors (in mm) for 2020.
**FIGURE S3.** Time series of correction factors for the Harquahala INA and McMullen Valley.
**FIGURE S4.** Mean actual and predicted groundwater (GW) pumping over the AMA/INA region for each year, with 2010–2020 being validation or test years. Here, we replace the annual discharge data from the Parker Dam with the data from Lee's Ferry (Glen Canyon Dam).
**FIGURE S5.** Random Forest feature importances for the ten predictors for each of the three different splitting strategies‐ spatial (S), spatiotemporal (ST), and temporal (T). We observe that the spatially static predictors (WD, AGRI, URBAN, SW, CC, and AD) receive higher importance than the spatio‐temporal ones. Moreover, the feature importances are similar for each of the three splits.
**FIGURE S6.** Histogram showing the standardized residuals (temporal data splitting strategy) restricted within the [−2, 2] interval (the red line represents the Gaussian probability density function). Here, we have removed standardized residuals which are exactly 0 (82% of the standardized residuals) for appropriately showing the distribution.
**FIGURE S7.** The alfalfa acreage in the Harquahala Valley, Arizona obtained from the USDA‐NASS cropland data layer (CDL) product for 2008, 2010, 2015, and 2020. We notice that that the acreage has almost doubled between 2008 and 2020.
**FIGURE S8.** Map of Arizona showing the mean sediment thickness for each groundwater basin from 2010–2020 at 2 km resolution where the mean is taken over regions having TPGW ≥100 mm.
**TABLE S1.** Error metrics (rounded to 2 decimal places) over the AMA/INA region for the temporal data splitting strategy wherein we use 2002–2009 for training and 2010–2020 for testing the model, respectively.

## Data Availability

The entire project source code is publicly available at https://github.com/montimaj/HydroSAR.
