# Supplementary material for: Advancing remote sensing and machine learning‐driven frameworks for groundwater withdrawal estimation in Arizona: Linking land subsidence to groundwater withdrawals
Source: Hydrol Process. 2022 Nov 14;36(11):e14757. doi: 10.1002/hyp.14757 (PMC9828199; doi:10.1002/hyp.14757)
Supplement: Supplementary file 1 — FIGURE S1. Canal buffer map showing features from the NHD that were coded as canals. Here, we created a 1 km buffer around each of these features, and dissolved them so that overlapping features were combined into one large buffered region. FIGURE S2. Alfalfa‐based postprocessing correction factors (in mm) for 2020. FIGURE S3. Time series of correction factors for the Harquahala INA and McMullen Valley. FIGURE S4. Mean actual and predicted groundwater (GW) pumping over the AMA/INA region for each year, with 2010–2020 being validation or test years. Here, we replace the annual discharge data from the Parker Dam with the data from Lee's Ferry (Glen Canyon Dam). FIGURE S5. Random Forest feature importances for the ten predictors for each of the three different splitting strategies‐ spatial (S), spatiotemporal (ST), and temporal (T). We observe that the spatially static predictors (WD, AGRI, URBAN, SW, CC, and AD) receive higher importance than the spatio‐temporal ones. Moreover, the feature importances are similar for each of the three splits. FIGURE S6. Histogram showing the standardized residuals (temporal data splitting strategy) restricted within the [−2, 2] interval (the red line represents the Gaussian probability density function). Here, we have removed standardized residuals which are exactly 0 (82% of the standardized residuals) for appropriately showing the distribution. FIGURE S7. The alfalfa acreage in the Harquahala Valley, Arizona obtained from the USDA‐NASS cropland data layer (CDL) product for 2008, 2010, 2015, and 2020. We notice that that the acreage has almost doubled between 2008 and 2020. FIGURE S8. Map of Arizona showing the mean sediment thickness for each groundwater basin from 2010–2020 at 2 km resolution where the mean is taken over regions having TPGW ≥100 mm. TABLE S1. Error metrics (rounded to 2 decimal places) over the AMA/INA region for the temporal data splitting strategy wherein we use 2002–2009 for training and 2010–2020 for testing th [file HYP-36-0-s001.pdf]

Supplementary Information for

Advancing Remote Sensing and Machine Learning-Driven Frameworks for Groundwater Withdrawal Estimation in Arizona: Linking Land Subsidence to Groundwater Withdrawals

Majumdar et al., 2022

The supplementary information contains eight figures and a table referenced from the text. For a description of the data sources and the calculations used for Supplementary Figures 1-8 and Supplementary Table 1, see Methodology, Results and Analyses, and Discussion sections.

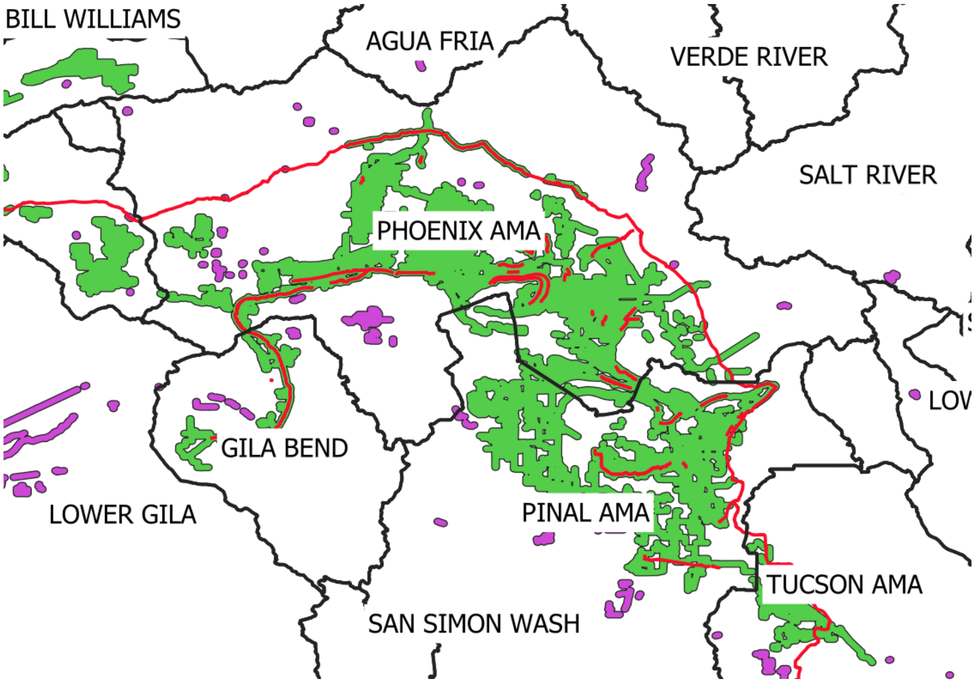

Supplementary Figure 1. Canal buffer map showing features from the NHD that were coded as canals. Here, we created a 1 km buffer around each of these features, and dissolved them so that overlapping features were combined into one large buffered region.

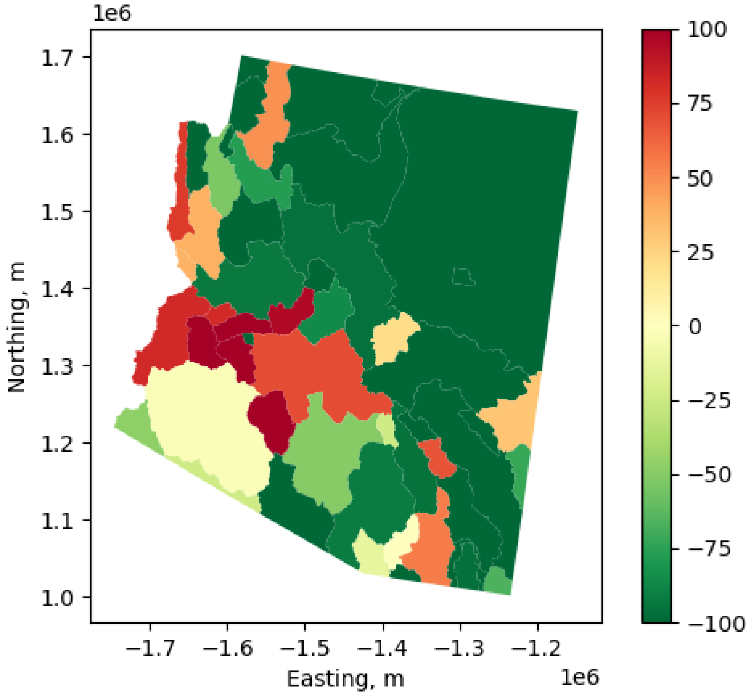

Supplementary Figure 2. Alfalfa-based postprocessing correction factors (in mm) for 2020.

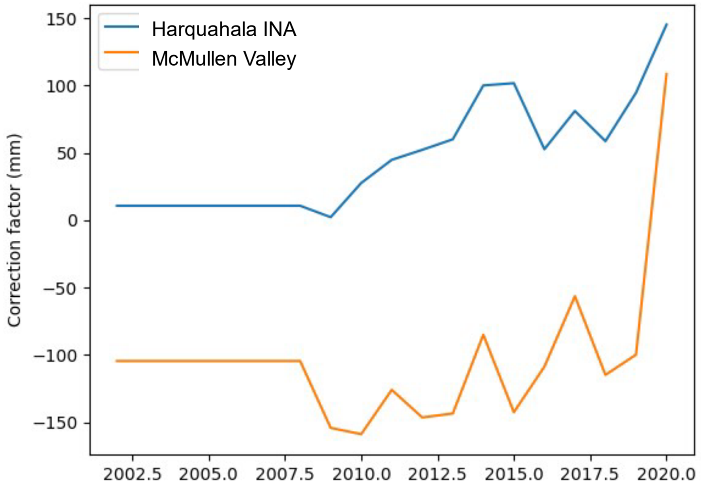

Supplementary Figure 3. Time series of correction factors for the Harquahala INA and McMullen Valley.

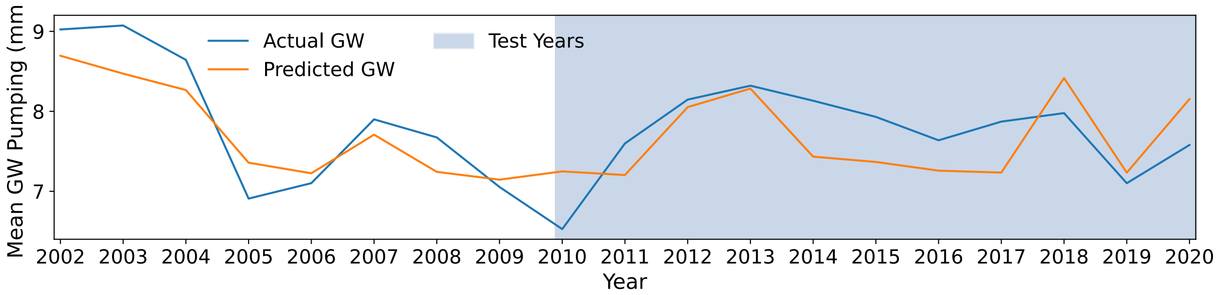

Supplementary Figure 4. Mean actual and predicted groundwater (GW) pumping over the AMA/INA region for each year, with 2010-2020 being validation or test years. Here, we replace the annual discharge data from the Parker Dam with the data from Lee's Ferry (Glen Canyon Dam).

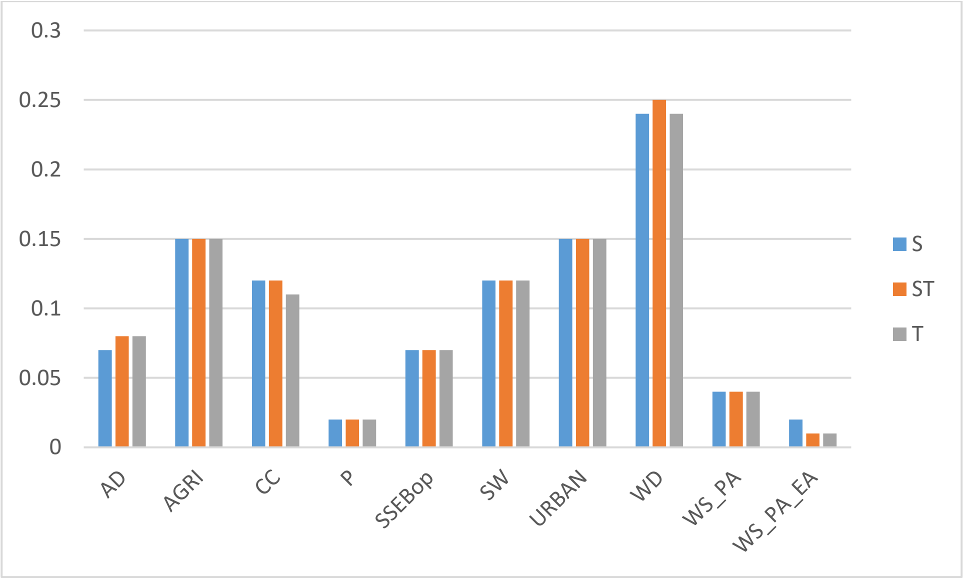

Supplementary Figure 5. Random Forest feature importances for the ten predictors for each of the three different splitting strategies- spatial (S), spatiotemporal (ST), and temporal (T). We observe that the spatially static predictors (WD, AGRI, URBAN, SW, CC, and AD) receive higher importance than the spatio-temporal ones. Moreover, the feature importances are similar for each of the three splits.

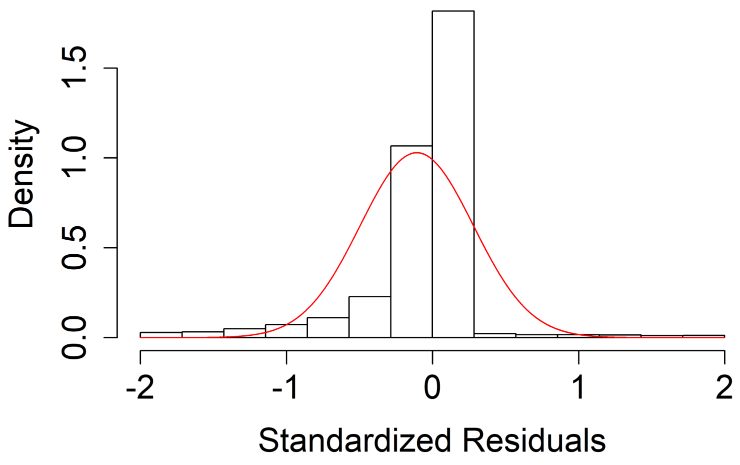

Supplementary Figure 6. Histogram showing the standardized residuals (temporal data splitting strategy) restricted within the [-2, 2] interval (the red line represents the Gaussian probability density function). Here, we have removed standardized residuals which are exactly 0 (82% of the standardized residuals) for appropriately showing the distribution.

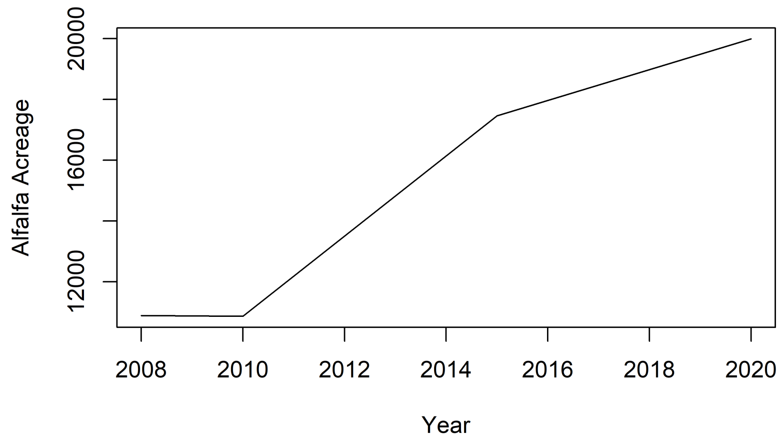

Supplementary Figure 7. The alfalfa acreage in the Harquahala Valley, Arizona obtained from the USDA-NASS cropland data layer (CDL) product for 2008, 2010, 2015, and 2020. We notice that that the acreage has almost doubled between 2008 and 2020.

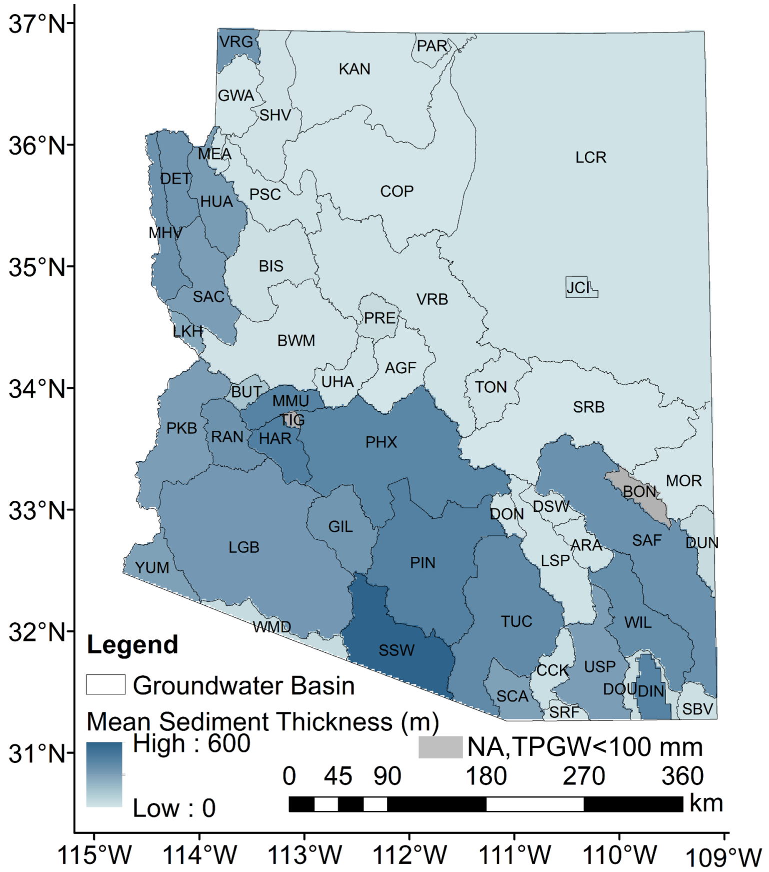

Supplementary Figure 8. Map of Arizona showing the mean sediment thickness for each groundwater basin from 2010-2020 at 2 km resolution where the mean is taken over regions having TPGW ≥ 100 mm.

Supplementary Table 1. Error metrics (rounded to 2 decimal places) over the AMA/INA region for the temporal data splitting strategy wherein we use 2002-2009 for training and 2010-2020 for testing the model, respectively.

Data	Year	R^2	NRMSE	NMAE
Train	2002	0.87	1.46	0.4
Train	2003	0.9	1.21	0.35
Train	2004	0.92	1.14	0.32
Train	2005	0.89	1.4	0.4
Train	2006	0.9	1.37	0.38
Train	2007	0.92	1.19	0.33
Train	2008	0.91	1.27	0.37
Train	2009	0.89	1.42	0.41
Test	2010	0.78	2.11	0.59
Test	2011	0.78	2.05	0.55
Test	2012	0.78	1.92	0.51
Test	2013	0.74	2.13	0.57
Test	2014	0.7	2.28	0.61
Test	2015	0.7	2.28	0.62
Test	2016	0.64	2.56	0.65
Test	2017	0.66	2.43	0.64
Test	2018	0.66	2.44	0.66
Test	2019	0.61	2.69	0.72
Test	2020	0.61	2.73	0.74
